# DGAT1 regulates macrophage pyroptosis during *Klebsiella pneumoniae* infection

**DOI:** 10.1038/s41420-026-03272-y

**Published:** 2026-08-11

**Authors:** Hui-Jung Jung, Seong Hun Jeong, Sang-Hee Lee, Hyowon Hong, Yong-Uk Lee, Miri Hyun, Ji Yeon Lee, Won-Ki Baek, Jin Kyung Kim, Hyun ah Kim

**Affiliations:** 1 Department of Microbiology, Keimyung University School of Medicine, Daegu, South Korea; 2 Department of Infectious Diseases, Keimyung University Dongsan Hospital, Keimyung University School of Medicine, Daegu, South Korea; 3 Center for Bio-imaging & Translational Research (104-Dong), Korea Basic Science Institute, Cheongju, South Korea; 4 Department of Chemistry, Hanyang University, Seoul, South Korea; 5 Department of Anatomy, Keimyung University School of Medicine, Daegu, South Korea

**Keywords:** Infection, Cell death and immune response

## Abstract

*Klebsiella pneumoniae* (Kp) is a Gram-negative opportunistic pathogen that causes severe infections, including pneumonia and sepsis, yet the molecular mechanisms governing macrophage cell death during Kp infection are incompletely understood. Here, we identify diacylglycerol acyltransferase-1 (DGAT1) as a critical regulator of macrophage pyroptosis during infection with a classical Kp (cKp) isolate. cKp infection induced lipid droplet (LD) accumulation in murine macrophages, whereas pharmacological inhibition or genetic suppression of DGAT1 attenuated LD formation and pyroptotic cell death. Mechanistically, DGAT1-dependent LD accumulation was associated with lysosomal dysfunction and mitochondrial reactive oxygen species (ROS) production, which together facilitated NLRP3 inflammasome activation and pyroptosis. Furthermore, LD accumulation, ROS production, and mTOR signaling were mutually interconnected, as inhibition of any one component attenuated the others, collectively sustaining pyroptotic signaling during cKp infection. Small RNA sequencing further identified miR-155-5p as a ROS-dependent microRNA up-regulated during cKp infection, which promoted LD accumulation in a DGAT1-dependent manner and contributed to pyroptotic cell death. Collectively, these findings identify DGAT1 as a key immunometabolic regulator of macrophage pyroptosis and suggest that targeting DGAT1 may represent a potential therapeutic strategy to mitigate Kp infection-induced macrophage pyroptosis.

## Introduction

Classical *Klebsiella pneumoniae* (cKp) is an opportunistic pathogen that causes a wide spectrum of nosocomial infections, including pneumonia, bloodstream infections, urinary tract infections, and device-associated infections, particularly in immunocompromised hosts and critically ill patients. Globally, the clinical burden of cKp infections has escalated with the increasing prevalence of multidrug-resistant (MDR) and carbapenem-resistant *K. pneumoniae* (CRKP) strains. According to the World Health Organization and the Centers for Disease Control, CRKP is now recognized as a critical priority pathogen owing to its limited treatment options and high associated mortality, often exceeding 40% in bloodstream infections [1, 2]. Recent epidemiological surveillance reports have shown that cKp strains remain the dominant lineage in healthcare-associated infections across Asia, Europe, and North America, although the proportion of hypervirulent or hybrid strains is also gradually increasing [1, 2]. These trends underscore the urgent need for an improved understanding of host–pathogen interactions in cKp infections to guide the development of novel therapeutic strategies.

Diacylglycerol O-acyltransferase-1 (DGAT1) is a key enzyme that catalyzes the conversion of diacylglycerol (DAG) and acyl-CoA into triacylglycerol (TG), which is stored within lipid droplets (LD) [3]. In humans, DGAT1 has emerged as a therapeutic target for metabolic disorders such as obesity [4], type 2 diabetes [5], and metabolic dysfunction-associated steatotic liver disease [6], with several DGAT1 inhibitors currently under clinical investigation [7–9]. Although DGAT1 is best known for its role in lipid metabolism and energy homeostasis, it is also involved in host–pathogen interactions and the progression of certain infectious diseases [10, 11]. Our previous study demonstrated that the inhibition of DGAT1 reduces LD formation, thereby limiting hypervirulent Kp (hvKp) growth within host cells [12]. Additionally, during *Mycobacterium tuberculosis* infection, host macrophages accumulate LDs, which serve as nutrient reservoirs that support bacterial survival and persistence [13].

Although traditionally studied for its metabolic functions, accumulating evidence suggests that DGAT1 plays a critical role in modulating various forms of programmed cell death, including apoptosis and ferroptosis [14, 15]. For example, DGAT1 can exert a protective effect against palmitic acid–induced apoptosis by channeling toxic lipid intermediates into neutral TG, thereby reducing endoplasmic reticulum (ER) stress and proinflammatory responses [16]. Conversely, under certain pathological conditions, dysregulation or overactivation of DGAT1 may contribute to cellular stress and death. For example, excessive TG synthesis and LD accumulation can disrupt organelle function, leading to the activation of pro-death signaling cascades [17]. Therefore, understanding the context-dependent role of DGAT1 in cell death pathways may offer new therapeutic insights, particularly in the settings of metabolic disease and infection.

Reactive oxygen species (ROS) function as intracellular signaling molecules and mediators of oxidative damage [18]. When produced in excess, ROS can induce oxidative stress, leading to structural and functional disruption of proteins, lipids, and nucleic acids [19, 20]. Importantly, ROS are now recognized not only as effectors of cellular injury but also as upstream regulators of gene expression, particularly through the modulation of microRNAs (miRNAs)—small non-coding RNAs that play pivotal roles in post-transcriptional gene regulation [21, 22]. ROS-regulated miRNAs serve as critical molecular switches in controlling cell death pathways [23]. For instance, miR-34a, a well-characterized ROS-inducible miRNA, is transcriptionally activated by p53 in response to DNA damage and oxidative stress. miR-34a promotes apoptosis by targeting anti-apoptotic genes, such as Bcl-2 and sirtuin 1, thus amplifying pro-death signaling [24].

In this study, we identify DGAT1 as a regulator of macrophage pyroptosis during cKp infection. DGAT1 inhibition suppressed cKp-induced pyroptosis by limiting lysosomal dysfunction and mitochondrial ROS production, thereby attenuating NLRP3 inflammasome activation. We further demonstrate that LD accumulation, ROS production, and mTOR signaling are mutually interconnected during cKp infection, and that miR-155-5p functions as a ROS-dependent microRNA that promotes LD accumulation and pyroptotic cell death in a DGAT1-dependent manner.

## Results

### DGAT1 inhibition reduces LD formation induced by cKp in macrophages

**Fig. 1:**
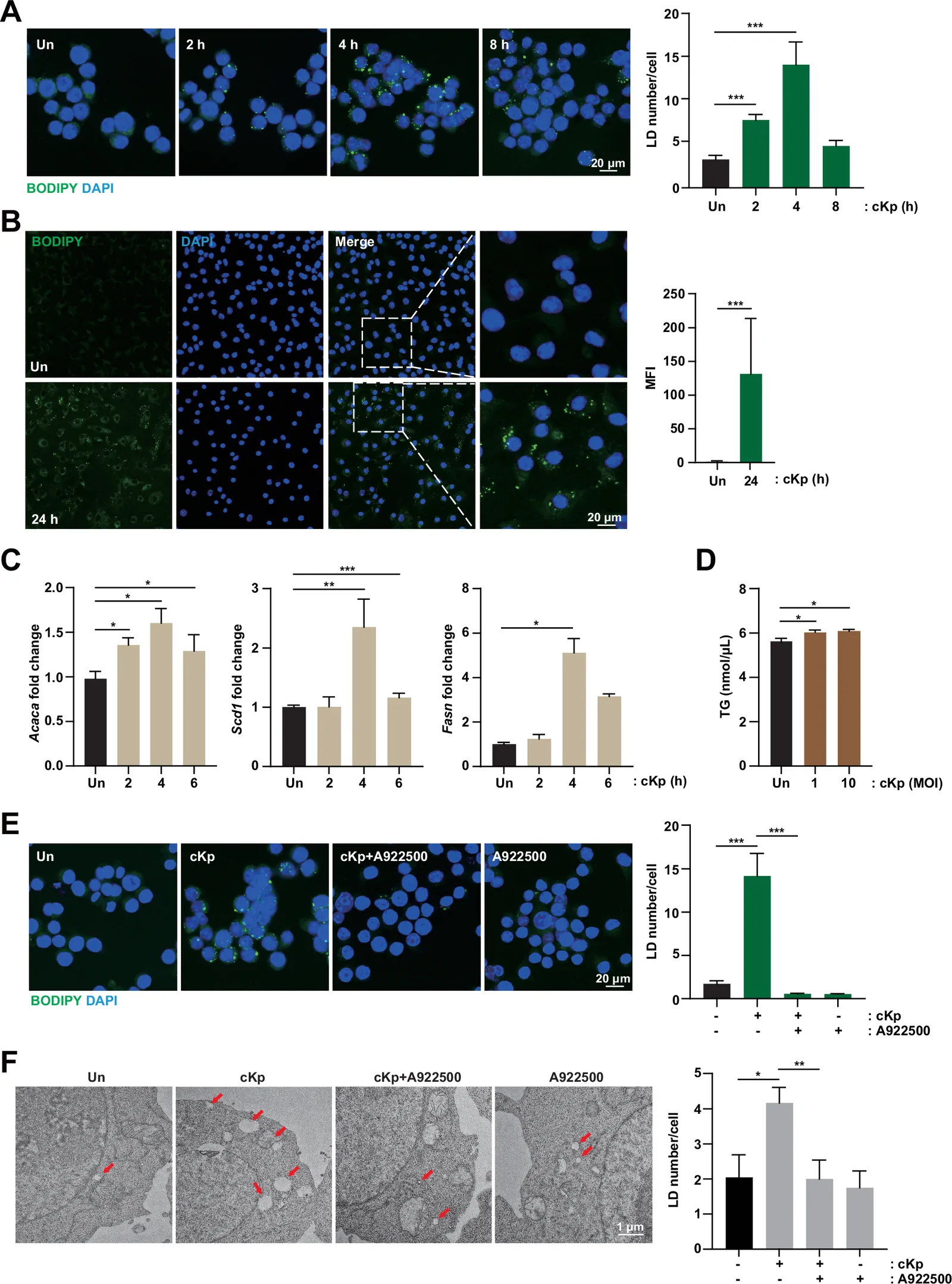
DGAT1 inhibition reduces LD formation in macrophages during cKp infection. **A** RAW264.7 cells were infected with cKp strain (MOI 1) for 2, 4, or 8 h, and stained with BODIPY 493/503 (green) and DAPI (for nuclei). Representative confocal images are shown. Scale bar, 20 μm (left). Quantification of LD number per cell is shown (right). **B** BMDMs were infected with cKp (MOI 10) for 24 h and stained with BODIPY 493/503 (green) and DAPI. Representative confocal images are shown. Scale bar, 20 μm (left). Mean fluorescence intensity (MFI) of BODIPY 493/503 is shown (right). **C** RAW264.7 cells were infected with cKp (MOI 1) for the indicated times, and the mRNA levels of lipogenic genes (*Acaca*, *Scd1*, and *Fasn*) were measured by qRT-PCR. **D** RAW264.7 cells were infected with cKp strain (MOI 1 or 10) for 4 h and triglycerides (TG) levels were measured using an enzymatic assay kit. **E** RAW264.7 cells were pretreated with A922500 (10 μM) for 1 h and then infected with cKp (MOI 1) for 4 h. LD formation was analyzed by confocal microscopy using BODIPY 493/503 staining and DAPI (for nuclei). **F** Transmission electron microscopy (TEM) images showing reduced LD formation in A922500-pretreated RAW264.7 cells following cKp infection (MOI 1, 4 h). Scale bar, 1 μm. Red arrows indicate LD. All quantitative data are expressed as the mean ± standard deviation of three independent experiments (*n* = 3). Un uninfected, LDs lipid droplets. *P < 0.05, **P < 0.01, and ***P < 0.001.

Based on our previous finding that clinical hvKp strains induce LD formation in murine macrophages [12], we investigated whether cKp infection results in a comparable response. LD formation was assessed in RAW264.7 cells and BMDMs using the fluorescent dye BODIPY 493/503, and cKp infection was found to induce LD accumulation (Fig. 1A, B). Moreover, cKp infection significantly increased the expression of genes related to fatty acid synthesis, including acetyl-CoA carboxylase alpha (*Acaca*), stearoyl-CoA 9-desaturase 1 (*Scd1*), and fatty acid synthase (*Fasn*) (Fig. 1C). Consistent with these changes, cKp infection also increased intracellular TG levels in macrophages (Fig. 1D). Using confocal microscopy, we confirmed that pharmacological inhibition of DGAT1 with A922500 attenuated LD formation in RAW264.7 cells (Fig. 1E) and BMDMs (Supplementary Fig. 1). Electron microscopy (EM) further revealed that A922500 treatment reduced cKp-induced LD formation in RAW264.7 cells (Fig. 1F). These findings suggested that cKp infection induces the synthesis of TG and the formation of LD in murine macrophages via a DGAT1-dependent mechanism.

### DGAT1 inhibition attenuates cKp-induced pyroptosis in macrophages

**Fig. 2:**
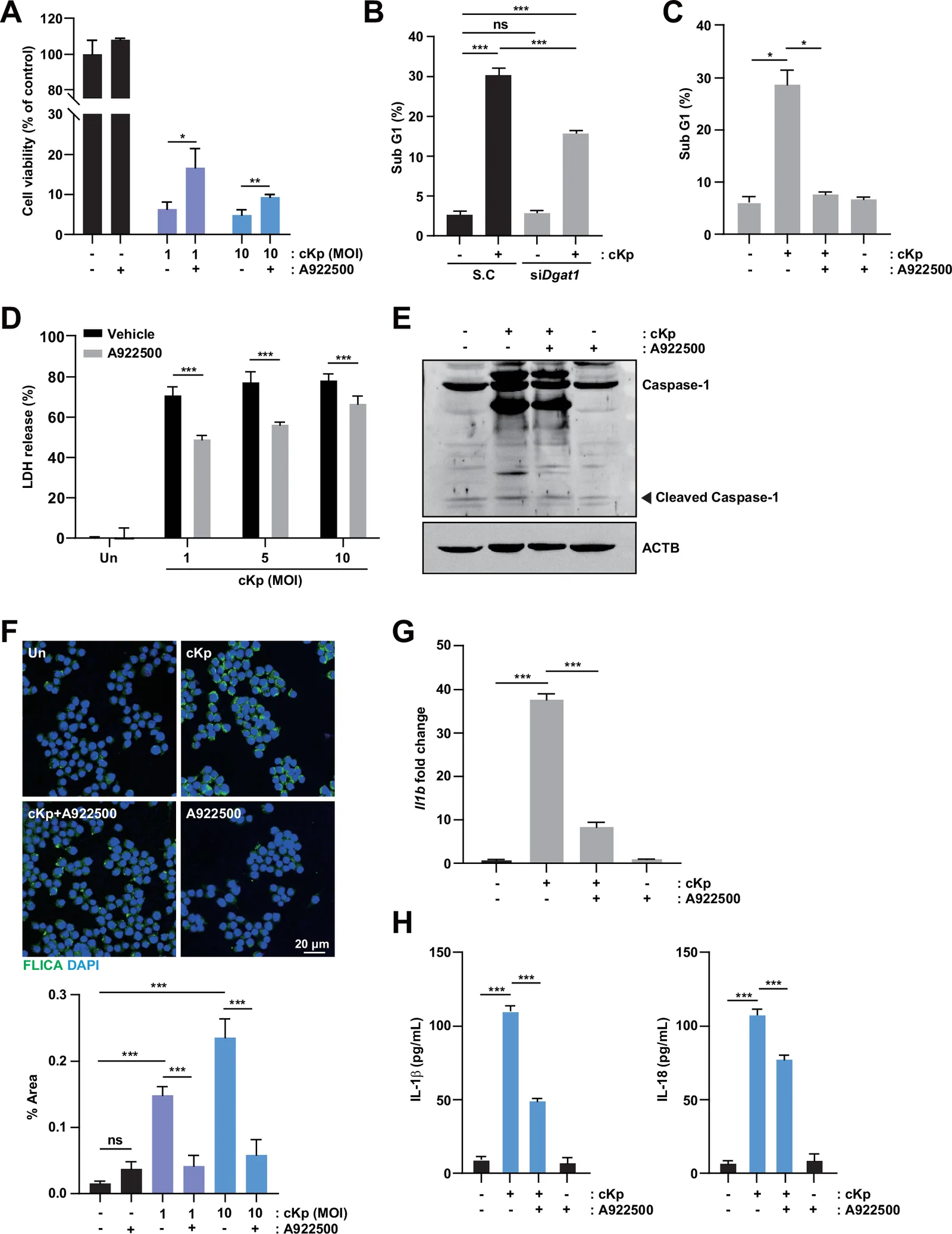
DGAT1 inhibition suppresses pyroptosis in cKp-infected macrophages. **A** RAW264.7 cells were pretreated with A922500 (10 μM) for 1 h, followed by infection with cKp (MOI 1 or 10) for 16 h. Cell viability was assessed using a trypan blue exclusion assay. **B** RAW264.7 cells were transfected with scrambled control (S.C) or *Dgat1*-specific siRNA for 24 h, followed by infection with cKp for 8 h. Cell death was quantified by PI staining-based flow cytometry. **C** RAW264.7 cells were pretreated with A922500 (10 μM) for 1 h and then infected with cKp (MOI 1) for 16 h. Cells were stained with PI, and the sub-G1 population was quantified by flow cytometry. **D** BMDMs were pretreated with A922500 (10 μM) for 1 h and then infected with cKp at the indicated MOIs. Cell death was quantified by measuring lactate dehydrogenase (LDH) release. **E** RAW264.7 cells were incubated with or without A922500 (10 μM) for 1 h and then infected with cKp (MOI 1) deletion deletion and incubated for 16 h. Cells were lysed and subjected to immunoblotting using antibodies against caspase-1 and ACTB. ACTB was used as a loading control. **F** RAW264.7 cells were infected with cKp (MOI 1 or 10) for 8 h, and cells were stained with FAM-FLICA for 1 h. Nuclei were stained with DAPI. Representative confocal images are shown. Scale bar, 20 μm (top). FAM-FLICA–positive area was quantified (bottom). **G** RAW264.7 cells were incubated with or without A922500 (10 μM) for 1 h and then infected with cKp (MOI 1) for 4 h. The mRNA expression levels of *Il1b* were measured using qRT-PCR. **H** BMDMs were pretreated with A922500 (10 μM) for 1 h and then infected with cKp (MOI 10) for 4 h. IL-1β and IL-18 levels in cell culture supernatants were determined by ELISA. All quantitative data are expressed as the mean ± SD of three independent experiments (*n* = 3). Un uninfected, ns not significant, S.C scrambled control. *P < 0.05, **P < 0.01, ***P < 0.001.

Our previous study demonstrated that cKp infection induces pyroptosis in murine macrophages [25] and that inhibition of LD formation is critical for controlling the intracellular survival of hvKp strains [12]. Based on these findings, we investigated whether the modulation of LD formation influences cell fate during cKp infection. The reduction in cell viability caused by cKp infection was restored by treatment with DGAT1 inhibitors (A922500 and T863) in RAW264.7 cells (Fig. 2A and Supplementary Fig. 2A). To determine whether DGAT1 influences cell survival, we used small interfering RNA (siRNA) to knock down *Dgat1* expression (Supplementary Fig. 2B). *Dgat1* knockdown rescued cell viability during cKp infection (Fig. 2B and Supplementary Fig. 2C). Moreover, treatment with the DGAT1 inhibitor during cKp infection reduced the sub-G1 population, which reflects cells with fragmented DNA (Fig. 2C and Supplementary Fig. 2D), and decreased the elevated LDH release in BMDMs (Fig. 2D). Conversely, treatment with the lipolysis inhibitor atglistatin, which prevents LD breakdown, further increased LDH release in cKp-infected macrophages (Supplementary Fig. 2E). Taken together, these findings suggest that cKp-induced cell death is closely linked to intracellular lipid accumulation. To determine whether the cKp-induced cell death attenuated by DGAT1 inhibition involved caspase-1 activation, we examined caspase-1 expression (Fig. 2E) and activity (Fig. 2F) by western blot analysis and confocal microscopy, respectively. DGAT1 inhibition attenuated the cKp-induced upregulation of caspase-1 expression and activity (Fig. 2E, F). Furthermore, DGAT1 inhibition reduced the mRNA expression of interleukin (*Il*)*1b* (Fig. 2G) and the secretion of IL-1β and IL-18 (Fig. 2H) induced by cKp infection. These data indicated that DGAT1 inhibition reduced cKp-induced pyroptosis in murine macrophages.

### DGAT1 inhibition suppresses NLRP3-mediated pyroptosis during cKp infection

**Fig. 3:**
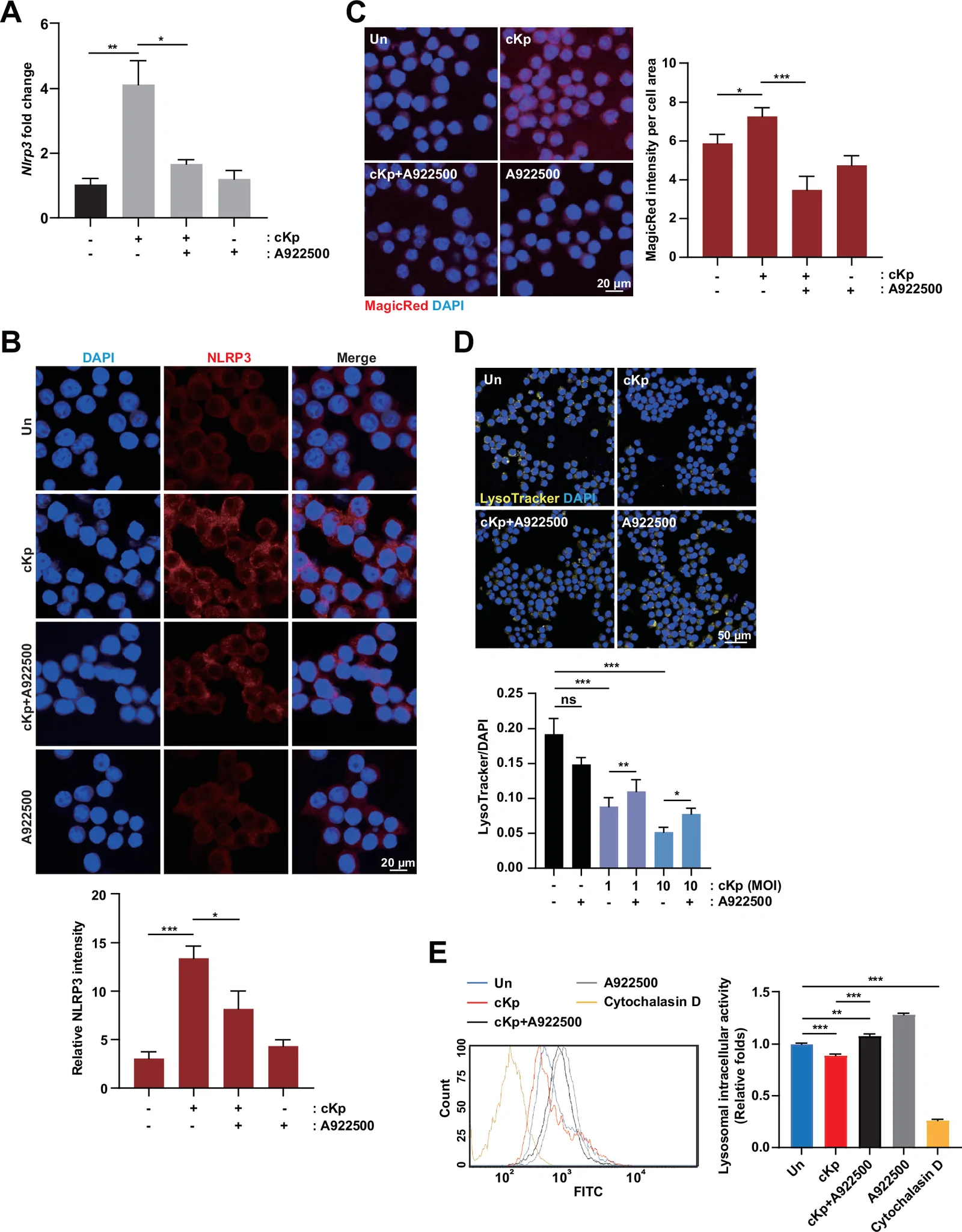
DGAT1 inhibition alleviates NLRP3-mediated pyroptosis by preserving lysosomal integrity during cKp infection. RAW264.7 cells were incubated with or without A922500 (10 μM) for 1 h and then infected with cKp (MOI 1) for 8 h. **A** The mRNA levels of *Nlrp3* were measured by qRT-PCR. **B** Cells were stained for NLRP3 (red) and DAPI (blue, nuclei). Representative confocal images (top) and quantification of the relative NLRP3 intensity are shown (bottom). Scale bar, 20 μm. **C** RAW264.7 cells were incubated with or without A922500 (10 μM) for 1 h and then infected with cKp (MOI 1). Cathepsin B activity was assessed using MagicRed staining for 1 h, followed by confocal imaging. Nuclei were stained with DAPI. Representative confocal images (left) and quantification of MagicRed intensity per cell area are shown (right). Scale bar, 20 μm. **D** RAW264.7 cells were incubated with or without A922500 (10 μM) for 1 h and then infected with cKp (MOI 1 or 10). Lysosomal integrity was evaluated by LysoTracker Yellow HCK-123 staining followed by confocal imaging. Representative confocal images (top) and quantification of LysoTracker/DAPI ratio are shown (bottom). Scale bar, 50 μm. **E** RAW264.7 cells were incubated with or without A922500 (10 μM) for 1 h and then infected with cKp (MOI 1). Lysosomal integrity was evaluated by LysoTracker Yellow HCK-123 staining followed by flow cytometry. Representative flow cytometry histograms (left) and quantification of lysosomal intracellular activity are shown (right). Cytochalasin D was used as a negative control. All quantitative data are expressed as the mean ± standard deviation of the three independent experiments (*n* = 3). Un uninfected, ns not significant. *P < 0.05, **P < 0.01, and ***P < 0.001.

To examine the role of DGAT1 inhibition in the modulation of NLRP3-dependent pyroptosis, we assessed the expression levels of NLRP3 mRNA and protein in RAW264.7 cells. Treatment with the DGAT1 inhibitor significantly reduced NLRP3 mRNA (Fig. 3A) and protein (Fig. 3B) levels during cKp infection. Our previous study demonstrated that cKp infection induces cathepsin B-mediated NLRP3 inflammasome activation [26]. We therefore investigated whether DGAT1 inhibition affects intracellular cathepsin B activity during cKp infection. DGAT1 inhibition indeed decreased cathepsin B activity (Fig. 3C). Given that cathepsin B is released from damaged lysosomes [26], we evaluated lysosomal integrity by measuring LysoTracker intensity and lysosomal activity. cKp infection led to a reduction in both parameters, indicating lysosomal dysfunction; however, treatment with the DGAT1 inhibitor A922500 during cKp infection restored lysosomal function, as evidenced by increased LysoTracker fluorescence intensity and lysosomal enzymatic activity (Fig. 3D, E). Collectively, these data suggest that DGAT1 inhibition alleviates NLRP3-mediated pyroptosis, at least in part by preserving lysosomal integrity during cKp infection.

### DGAT1 and mTOR signaling cooperatively regulate LD accumulation and pyroptosis during cKp infection

**Fig. 4:**
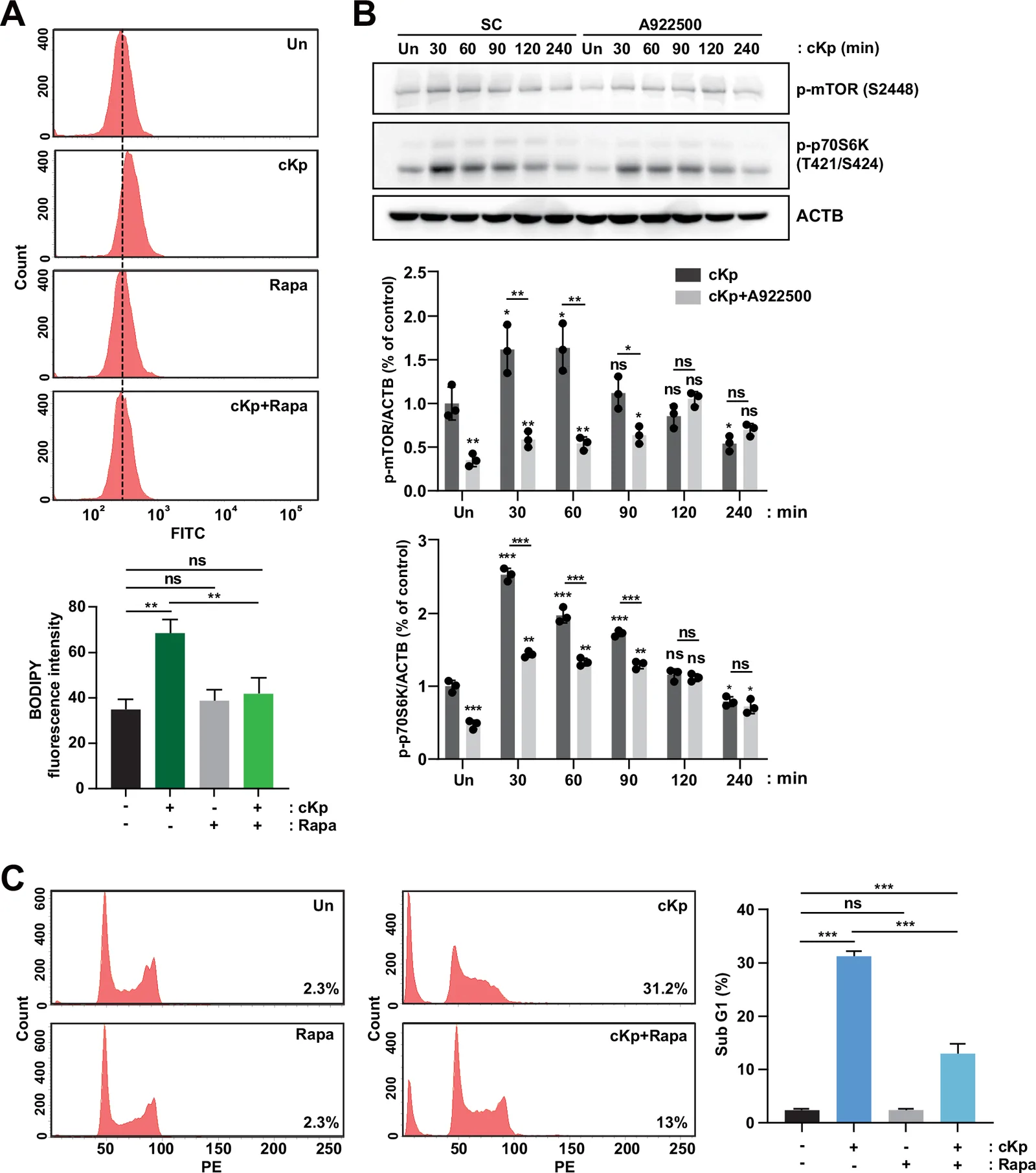
Rapamycin attenuates LD formation and restores cell viability, and DGAT1 inhibition reduces mTOR pathway phosphorylation during cKp infection. **A** RAW264.7 cells were pretreated with or without rapamycin (200 nM) for 30 min and then infected with cKp (MOI 10) and incubated for 4 h. LD formation was assessed by BODIPY 493/503 fluorescence intensity using flow cytometry. Representative flow cytometry histograms (top) and quantification are shown (bottom). **B** RAW264.7 cells were pretreated with or without A922500 (10 μM) for 1 h and then infected with cKp (MOI 10) for the indicated times. Cell lysates were subjected to immunoblotting using antibodies against p-mTOR (S2448), p-p70S6K (T421/S424), and ACTB as a loading control. Representative blots (top) and quantification of band intensity are shown (bottom). **C** RAW264.7 cells were pretreated with or without rapamycin (200 nM) for 30 min and then infected with cKp (MOI 10) and incubated for 16 h. The sub-G1 population was analyzed by flow cytometry. Representative flow cytometry histograms (left) and quantification are shown (right). All quantitative data are expressed as the mean ± SD of three independent experiments (*n* = 3). Un uninfected, SC solvent control, ns, not significant; Rapa, rapamycin. *P < 0.05, **P < 0.01, ***P < 0.001.

Previous studies have shown that mTOR plays a critical role in regulating cell survival [27]. In addition, mTOR activation exacerbates pyroptosis in MDBK cells, whereas treatment with rapamycin, an mTOR inhibitor, attenuates the expression of pyroptosis-associated proteins [28]. Therefore, we investigated whether DGAT1 inhibition affects mTOR-mediated pyroptosis during cKp infection. Rapamycin treatment attenuated LD formation during cKp infection (Fig. 4A), suggesting that mTOR signaling contributes to LD accumulation in this context. Rapamycin markedly reduced mTOR expression under these conditions (Supplementary Fig. 3). Furthermore, DGAT1 inhibition by A922500 reduced the phosphorylation of mTOR pathway–related proteins during cKp infection (Fig. 4B). Furthermore, rapamycin treatment restored cell viability impaired by cKp infection (Fig. 4C). These findings suggest that DGAT1 and mTOR signaling are functionally interconnected and that both contribute to LD accumulation and pyroptotic cell death during cKp infection.

### DGAT1 drives ROS-mediated pyroptosis via bidirectional crosstalk with mTOR signaling during cKp infection

**Fig. 5:**
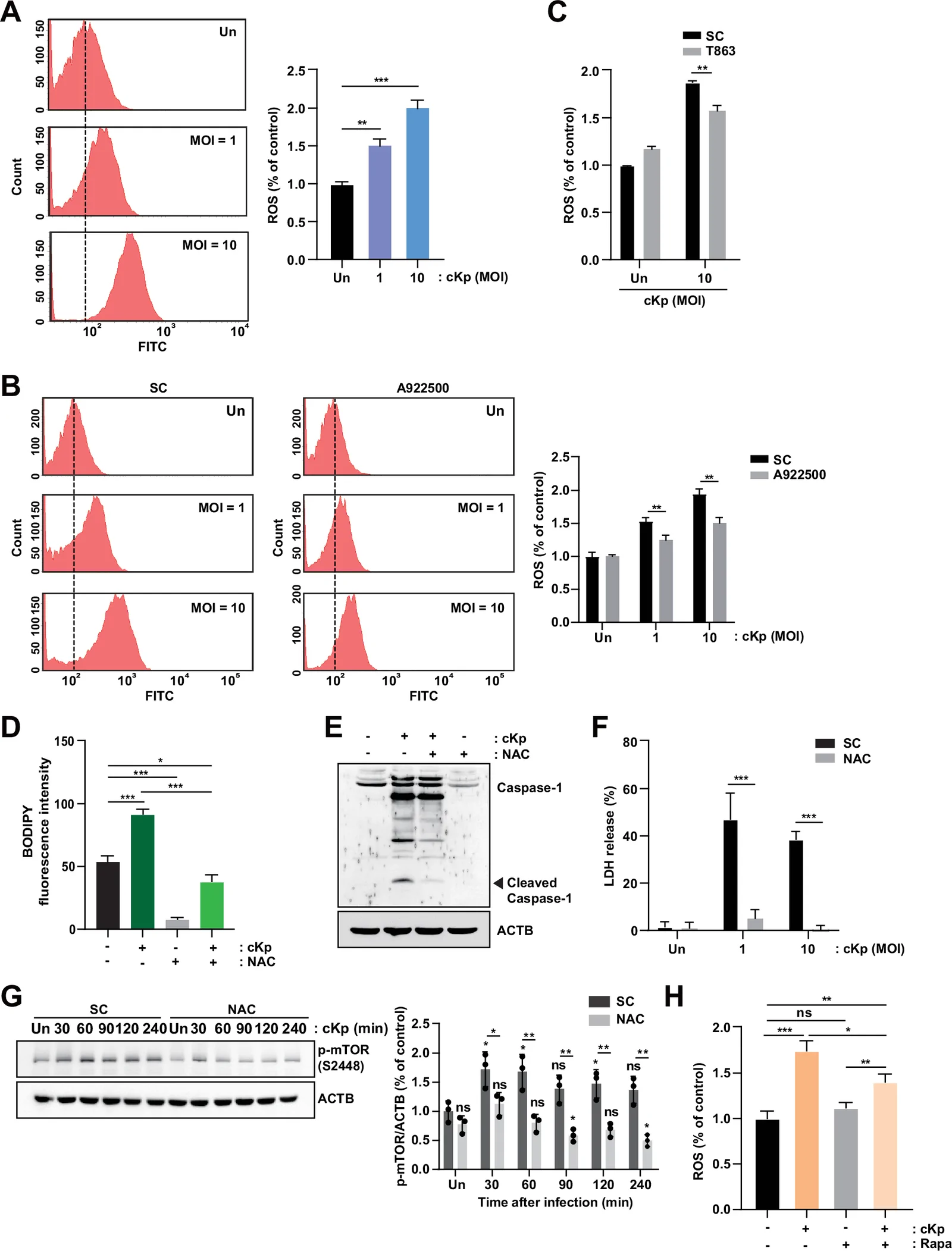
DGAT1 inhibition and ROS scavenging attenuate cKp-induced LD accumulation, pyroptosis, and mTOR–ROS bidirectional signaling. **A** RAW264.7 cells were infected with cKp at the indicated MOIs and incubated for 4 h. Intracellular ROS levels were measured by H₂DCFDA staining and flow cytometry. Representative flow cytometry histograms (left) and quantification are shown (right). **B** RAW264.7 cells were pretreated with A922500 (10 μM) for 1 h and then infected with cKp at the indicated MOIs and incubated for 4 h. Intracellular ROS levels were measured by H₂DCFDA staining and flow cytometry. **C** RAW264.7 cells were pretreated with T863 (5 μM) for 1 h and then infected with cKp (MOI 10) and incubated for 4 h. Intracellular ROS levels were measured by H₂DCFDA staining and flow cytometry. **D** RAW264.7 cells were pretreated with or without NAC (5 mM) for 1 h and then infected with cKp and incubated for 4 h. LD formation was assessed by BODIPY 493/503 fluorescence intensity using flow cytometry. RAW264.7 cells were pretreated with or without NAC (5 mM) for 1 h and then infected with cKp and incubated for 16 h. Caspase-1 expression was analyzed by immunoblotting, with ACTB as a loading control (**E**, MOI 1). Cell death was quantified by measuring LDH release (**F**, MOI 1 or 10). **G** RAW264.7 cells were pretreated with or without NAC (5 mM) for 1 h and then infected with cKp (MOI 10) for the indicated times. Cell lysates were subjected to immunoblotting using antibodies against p-mTOR (S2448), with ACTB as a loading control. Representative blots (left) and quantification are shown (right). **H** RAW264.7 cells were pretreated with or without rapamycin (200 nM) for 30 min and then infected with cKp and incubated for 4 h. Intracellular ROS levels were measured by H₂DCFDA staining and flow cytometry. All quantitative data are expressed as the mean ± SD of three independent experiments (*n* = 3). Un uninfected, ns not significant, SC solvent control, Rapa rapamycin. *P < 0.05, **P < 0.01, ***P < 0.001.

Excessive ROS production activates the NLRP3 inflammasome, thereby promoting pyroptosis [29]. To determine whether DGAT1 regulates pyroptosis through modulation of ROS during cKp infection, we first assessed intracellular ROS levels following infection. ROS production was significantly increased in an MOI-dependent manner upon cKp infection (Fig. 5A). Furthermore, pharmacological inhibition of DGAT1 using A922500 (Fig. 5B) or T863 (Fig. 5C) markedly reduced ROS production in both RAW264.7 cells and BMDMs (Supplementary Fig. 4A) during cKp infection. In addition, to further investigate whether DGAT1 inhibition affects mitochondrial ROS (mtROS) production, we measured mtROS levels using MitoSOX staining. A922500 treatment significantly reduced cKp-induced mtROS generation (Supplementary Fig. 4B), indicating that DGAT1-dependent LD accumulation contributes to mitochondrial dysfunction during cKp infection. To determine whether DGAT1-dependent oxidative stress contributes to lipid peroxidation during cKp infection, we assessed lipid peroxidation levels using C11-BODIPY 581/591. Although cKp infection significantly elevated lipid peroxidation levels, A922500 treatment did not affect them (Supplementary Fig. 4C). These results suggest that the DGAT1-dependent component of pyroptosis during cKp infection is not mediated through lipid peroxidation. To investigate the role of ROS in LD formation and pyroptosis, cells were treated with the ROS scavenger N-acetylcysteine (NAC). NAC treatment attenuated cKp-induced LD accumulation (Fig. 5D and Supplementary Fig. 4D). Importantly, NAC treatment markedly suppressed caspase-1 expression (Fig. 5E) and reduced LDH release (Fig. 5F) during cKp infection, supporting the critical role of ROS in pyroptotic cell death.

Next, to investigate the interplay between ROS and mTOR signaling during cKp infection, we treated macrophages with NAC or rapamycin. NAC treatment suppressed mTOR phosphorylation (Fig. 5G), whereas rapamycin treatment reduced ROS production during cKp infection (Fig. 5H), suggesting a bidirectional relationship between ROS and mTOR signaling. Collectively, these findings suggest that DGAT1 promotes ROS-dependent pyroptosis during cKp infection, potentially through reciprocal regulation between ROS and mTOR signaling.

### DGAT1 inhibition suppresses ROS-induced miR-155-5p expression to attenuate pyroptosis during cKp infection

**Fig. 6:**
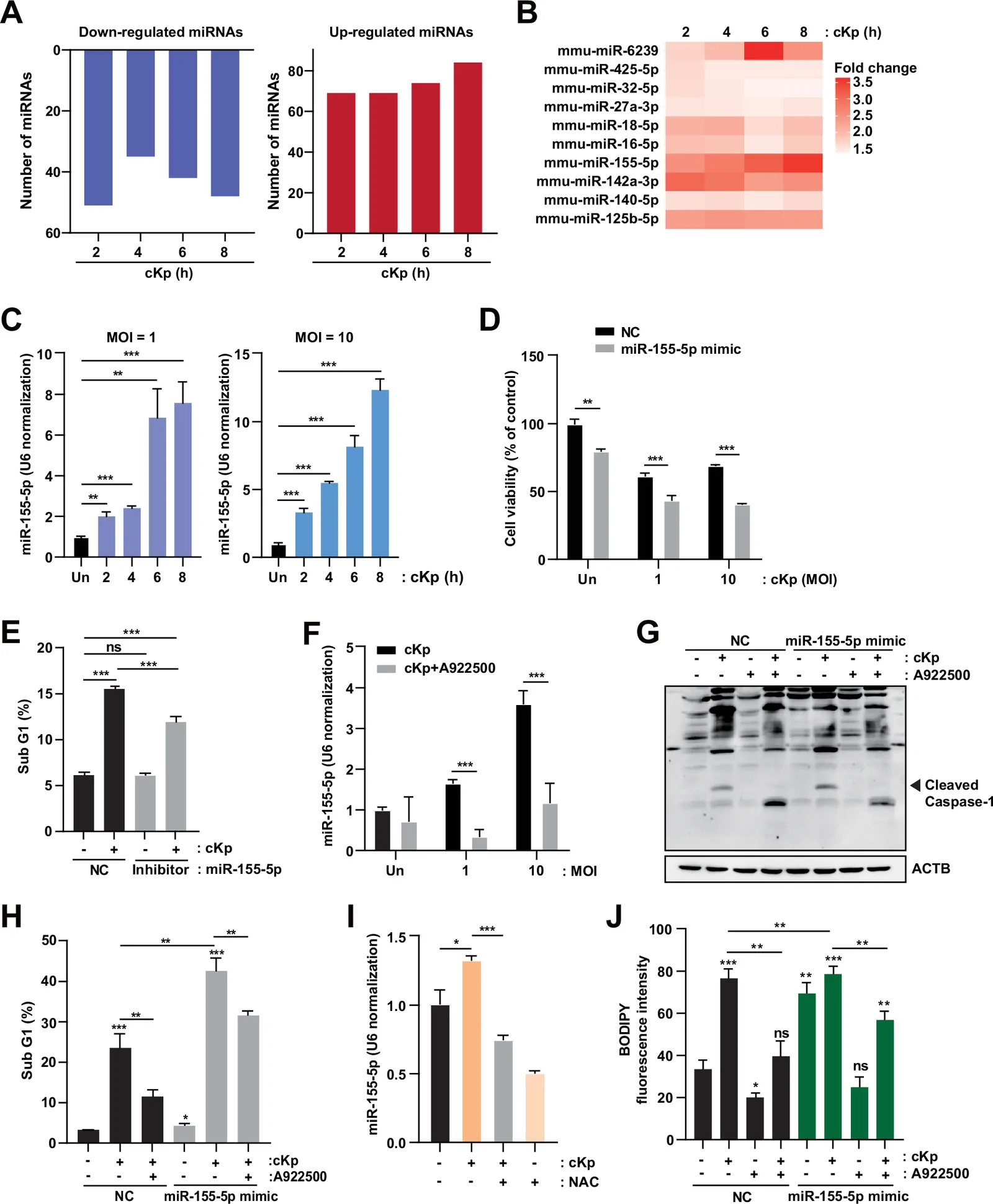
DGAT1 inhibition attenuates miR-155-5p expression and associated cell death. **A** The number of differentially expressed miRNAs in RAW264.7 cells infected with cKp (MOI 10) at the indicated time points (2, 4, 6, and 8 h) identified by small RNA sequencing. Down-regulated (left) and up-regulated miRNAs are shown (right). **B** Heatmap showing the fold change of up-regulated miRNAs following cKp infection at the indicated time points. **C** RAW264.7 cells were infected with cKp at an MOI of 1 or 10 for the indicated times. Expression levels of miR-155-5p were determined by qRT-PCR. **D** RAW264.7 cells were transfected with NC or miR-155-5p mimic (100 nM) for 24 h and then infected with cKp at the indicated MOIs and incubated for 8 h. Cell viability was assessed using a trypan blue exclusion assay. **E** RAW264.7 cells were transfected with NC or miR-155-5p inhibitor (100 nM) for 24 h and then infected with cKp (MOI 10) for 8 h. The sub-G1 population was analyzed by flow cytometry. **F** RAW264.7 cells were incubated with or without A922500 (10 μM) for 1 h and then infected with cKp at an MOI of 1 or 10 for 8 h. Expression levels of miR-155-5p were determined by qRT-PCR. **G** RAW264.7 cells were transfected with NC or miR-155-5p mimic (100 nM) for 24 h, pretreated with or without A922500 (10 μM) for 1 h, and then infected with cKp (MOI 1) and incubated for 16 h. Caspase-1 expression was analyzed by immunoblotting, with ACTB as a loading control. **H** RAW264.7 cells were transfected with NC or miR-155-5p mimic (100 nM) for 24 h, pretreated with or without A922500 (10 μM) for 1 h, and then infected with cKp (MOI 10) and incubated for 8 h. The sub-G1 population was analyzed by flow cytometry. **I** RAW264.7 cells were incubated with or without NAC (5 mM) for 1 h and then infected with cKp (MOI 1) and incubated for 8 h. Expression levels of miR-155-5p were determined by qRT-PCR. **J** RAW264.7 cells were transfected with NC or miR-155-5p mimic (100 nM) for 24 h, pretreated with or without A922500 (10 μM) for 1 h, and then infected with cKp (MOI 10) and incubated for 4 h. LD formation was assessed by BODIPY 493/503 fluorescence intensity using flow cytometry. All quantitative data are expressed as the mean ± standard deviation of three independent experiments (*n* = 3). Un uninfected, NC negative control, ns not significant. *P < 0.05, **P < 0.01, and ***P < 0.001.

To identify additional mechanisms by which DGAT1 regulates pyroptotic cell death, we performed small RNA sequencing analysis in cKp-infected RAW264.7 cells. To evaluate temporal changes in miRNA expression during infection, cells were infected with cKp for up to 8 h, and differentially expressed miRNAs were analyzed (Fig. 6A). Among the significantly up-regulated miRNAs, miR-155-5p exhibited a sustained increase in expression throughout the infection period (Fig. 6B). Previous studies have shown that miR-155 contributes to inflammatory cell death during bacterial infection [30, 31]. Therefore, we further investigated the role of miR-155-5p during cKp infection. cKp infection progressively increased miR-155-5p expression in both RAW264.7 cells (Fig. 6C) and BMDMs (Supplementary Fig. 5A). Functionally, transfection with an miR-155-5p mimic further reduced macrophage viability during cKp infection (Fig. 6D), whereas miR-155-5p inhibition restored cell viability (Fig. 6E and Supplementary Fig. 5B), suggesting that miR-155-5p contributes to cKp-induced cell death. The transfection efficiencies of the mimic and inhibitor were confirmed (Supplementary Fig. 5C, D). Notably, treatment with DGAT1 inhibitors reduced miR-155-5p expression in both RAW264.7 cells (Fig. 6F and Supplementary Fig. 5E) and BMDMs (Supplementary Fig. 5F). Consistent with this, A922500 treatment reduced miR-155-5p mimic–induced caspase-1 cleavage (Fig. 6G) and partially restored cell viability (Fig. 6H and Supplementary Fig. 5G) during cKp infection.

Because ROS is known to regulate miRNA expression [22], we next investigated whether ROS contributes to miR-155-5p induction during cKp infection. NAC treatment significantly reduced miR-155-5p expression (Fig. 6I). We next examined whether miR-155-5p contributes to LD formation. The miR-155-5p mimic increased LD accumulation under both basal and infected conditions; however, it was unable to rescue LD formation in A922500-treated infected macrophages (Fig. 6J and Supplementary Fig. 5H). These findings suggest that although miR-155-5p promotes LD formation, DGAT1 enzymatic activity is required for cKp-induced LD biogenesis. Collectively, these findings suggest that DGAT1 inhibition suppresses ROS-associated miR-155-5p expression, which may contribute to reduced pyroptotic cell death and enhanced macrophage survival during cKp infection.

### CRISPR/Cas9-mediated *Dgat1* disruption attenuates cell death, ROS production, and mTOR signaling during cKp infection

**Fig. 7:**
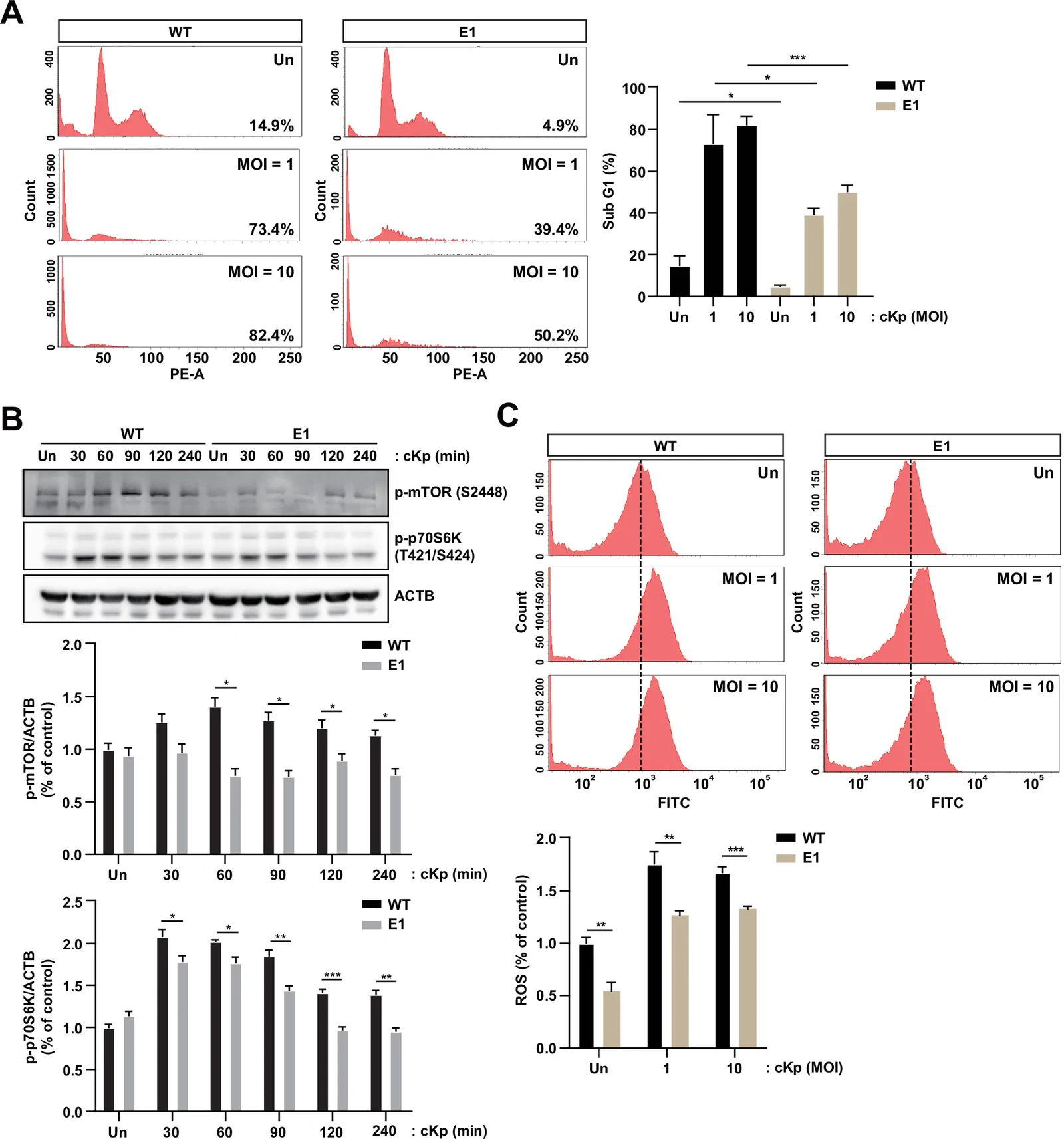
mTOR pathway mediates DGAT1-regulated cell survival during cKp infection. **A** RAW264.7 WT and *Dgat1* heterozygous knockout line (E1) cells were infected with cKp (MOI 1 or 10) for 16 h. Cells were stained with PI, and the sub-G1 population was quantified by flow cytometry. Representative flow cytometry histograms (left) and quantification are shown (right). **B** RAW264.7 WT and E1 cells were infected with cKp (MOI 10) for the indicated times. Cell lysates were subjected to immunoblotting using antibodies against p-mTOR (S2448), p-p70S6K (T421/S424), and ACTB as a loading control. Representative blots (top) and quantification of band intensity are shown (bottom). **C** RAW264.7 WT and E1 cells were infected with cKp (MOI 1 or 10) and intracellular ROS levels were measured 4 h post-infection using flow cytometry. ROS levels were quantified as mean fluorescence intensity. All quantitative data are expressed as the mean ± SD of three independent experiments (*n* = 3). Un uninfected. *P < 0.05, **P < 0.01, ***P < 0.001.

**Fig. 8:**
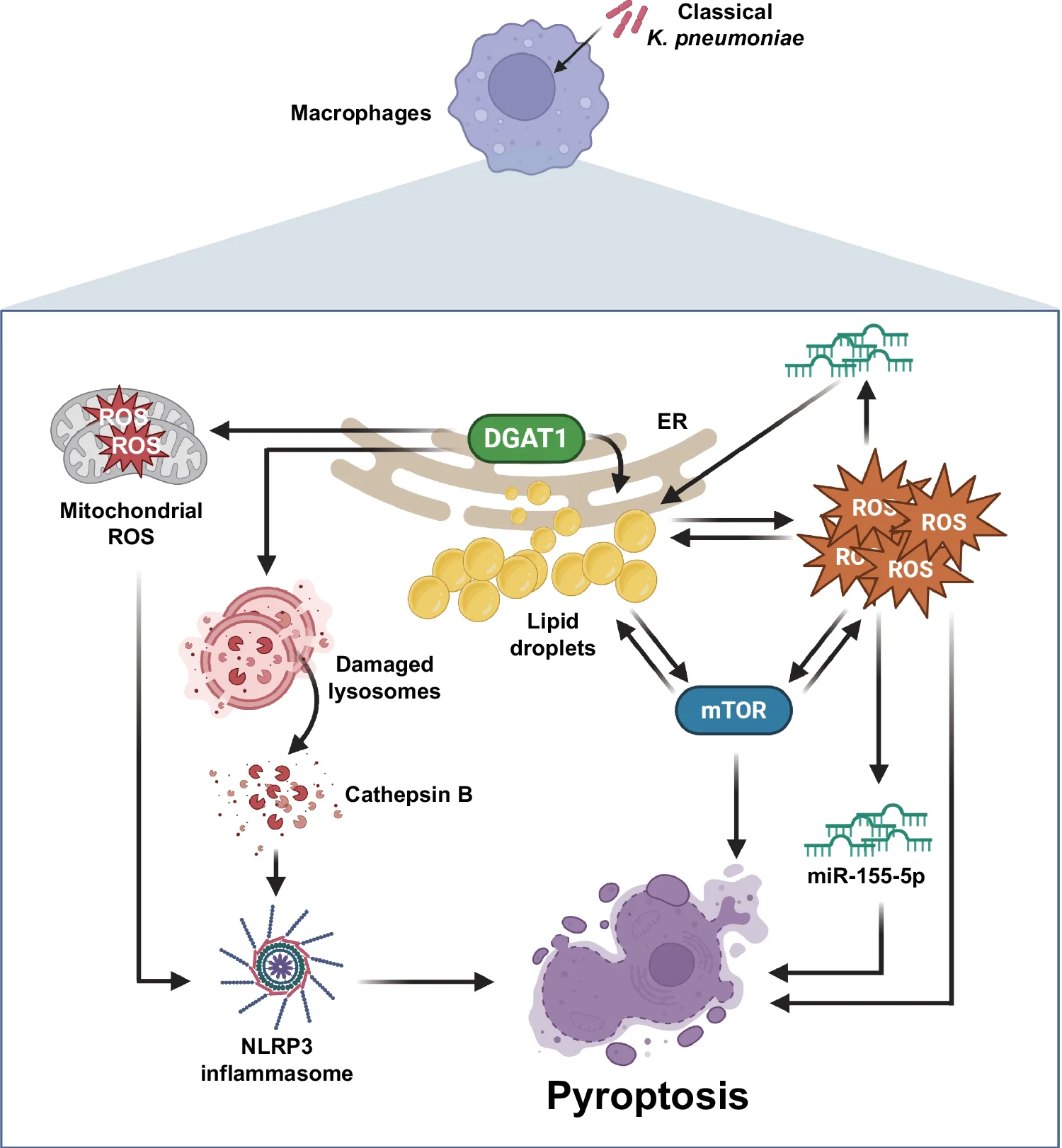
Schematic summary of the role of DGAT1 in macrophage responses to cKp infection. cKp infection promotes DGAT1-dependent LD biogenesis and mTOR pathway activation, which collectively drive ROS production, lysosomal damage, cathepsin B release, and miR-155-5p upregulation, ultimately culminating in pyroptotic macrophage death.

We generated a *Dgat1* heterozygous knockout cell line (*Dgat1*^+/–^) by targeting an early coding exon of *Dgat1* using the CRISPR/Cas9 system. A clone containing a 72-bp deletion in one allele and a wild-type (WT) allele in the other was selected, yielding a *Dgat1* heterozygous knockout line (E1). The mutation pattern was further confirmed by Sanger sequencing (Supplementary Fig. 6A). Immunoblot analysis demonstrated that DGAT1 protein expression was reduced in E1 cells compared with WT cells (Supplementary Fig. 6B). We next compared cKp-induced cell death between WT and E1 RAW264.7 cells. The proportion of cells in the sub-G1 phase was significantly lower in E1 cells than in WT cells (Fig. 7A). In addition, similar to pharmacological DGAT1 inhibition, the phosphorylation levels of mTOR pathway–related proteins were reduced in E1 cells during cKp infection (Fig. 7B). Flow cytometric analysis further revealed decreased ROS production in E1 cells (Fig. 7C). Collectively, these findings demonstrate that CRISPR/Cas9–mediated partial *Dgat1* deletion mitigates mTOR- and ROS-dependent cell death during cKp infection, corroborating the pharmacological inhibitor data. A summary diagram illustrating the proposed role of DGAT1 in macrophage pyroptosis during cKp infection is presented in Fig. 8.

## Discussion

DGAT1 is a key enzyme in TG synthesis and LD biogenesis. Although LDs were traditionally considered inert lipid storage organelles, recent studies have established that they function as dynamic immunometabolic platforms regulating inflammatory signaling, redox balance, and host–pathogen interactions during infection [30–32]. In macrophages, bacterial infection induces extensive lipid metabolic remodeling, including enhanced TG synthesis and LD accumulation, which can profoundly influence inflammatory responses and cell fate decisions [30, 33, 34]. Nevertheless, despite increasing interest in immunometabolism during bacterial infection, how host lipid metabolic pathways regulate macrophage survival during Kp infection has remained largely unclear.

In the present study, we demonstrate that DGAT1-dependent lipid metabolic remodeling critically regulates macrophage fate during infection with a cKp isolate. Unlike previous studies primarily focused on bacterial virulence factors or generalized inflammatory signaling pathways, our findings suggest that host lipid metabolism itself is an active determinant of pyroptotic cell death during cKp infection. This point is particularly important because excessive macrophage pyroptosis is thought to contribute not only to inflammatory tissue damage but also to bacterial dissemination during severe Kp infection. Our findings, therefore, suggest that DGAT1-dependent LD biogenesis is not simply a secondary consequence of infection but rather a key component of the pathogenic inflammatory response induced by cKp.

An important aspect of this study is the identification of DGAT1 as an upstream regulator of lysosome-associated pyroptotic signaling. Increasing evidence suggests that Kp modulates host cell death pathways in a strain-dependent manner [25, 35, 36]. In our previous study, we demonstrated that the clinical cKp isolate used here induces macrophage pyroptosis through activation of the cathepsin B–NLRP3–caspase-1 axis [25]. Building on these findings, the present study suggests that DGAT1-dependent LD accumulation contributes to lysosomal destabilization and cathepsin B release, thereby promoting NLRP3 inflammasome activation and pyroptotic cell death during cKp infection. This observation is particularly interesting because the relationship between LD biogenesis and lysosomal integrity during bacterial infection has not been clearly established. Our findings therefore support the concept that excessive LD accumulation in infected macrophages may contribute to lysosomal stress and amplify inflammasome-mediated inflammatory cell death.

ROS-associated inflammatory signaling also emerged as a major downstream consequence of DGAT1 activity during cKp infection. Excessive ROS production is a critical trigger of inflammasome activation and pyroptotic cell death [29, 37]. In our model, DGAT1 inhibition consistently reduced mtROS accumulation in RAW264.7 cells and BMDMs, whereas ROS scavenging attenuated LD formation, mTOR signaling, and pyroptosis-associated responses. Notably, lipid peroxidation, although elevated during cKp infection, was not affected by DGAT1 inhibition, indicating that DGAT1-mediated pyroptosis is driven by oxidative stress independently of lipid peroxidation. Rather than functioning as an isolated signaling event, ROS appear to operate at the interface of metabolic stress and inflammatory signaling during cKp infection. This interpretation is further supported by our observation that ROS scavenging suppressed mTOR activation and that rapamycin treatment reduced ROS production, suggesting reciprocal interactions between ROS and mTOR signaling in infected macrophages. Notably, DGAT1 inhibition by A922500 rapidly suppressed p-mTOR activation during cKp infection, suggesting that DGAT1 activity impacts mTOR signaling on a rapid timescale. Although mTOR signaling has been extensively studied in nutrient sensing and lipogenesis [38, 39], to the best of our knowledge, its regulation by DGAT1-dependent LD accumulation during bacterial infection has not been previously described, representing a previously uncharacterized mechanistic link between host lipid metabolism and nutrient-sensing mTOR signaling during cKp infection. Our findings therefore suggest that LD accumulation and mTOR signaling are functionally interconnected during cKp infection, as inhibition of either component attenuated the other, and that this bidirectional relationship collectively sustains ROS production and downstream pyroptotic signaling.

Another notable finding of this study is the identification of miR-155-5p as a ROS-dependent miRNA that contributes to pyroptotic cell death during cKp infection. miR-155 is a well-characterized inflammatory miRNA involved in macrophage activation and antibacterial responses [40, 41]. However, its role in regulating macrophage fate during cKp infection has not been clearly defined. Our findings suggest that miR-155-5p is induced downstream of ROS signaling during cKp infection and contributes to pyroptotic cell death. Importantly, miR-155-5p promoted LD accumulation in a DGAT1-dependent manner, suggesting that miR-155-5p functions within the DGAT1-ROS axis rather than as an independent signaling mediator. However, the precise molecular targets through which miR-155-5p contributes to pyroptotic cell death remain to be fully elucidated, and this represents an important direction for future investigation.

Several limitations should also be acknowledged. Although our findings demonstrate that DGAT1 regulates lysosomal dysfunction and ROS-associated pyroptotic signaling during cKp infection, the precise molecular mechanisms linking LD biogenesis to lysosomal destabilization remain unclear. In addition, although miR-155-5p contributed to pyroptotic cell death, the downstream target genes responsible for this phenotype were not identified. Finally, because our study primarily used in vitro macrophage infection models, future in vivo studies will be necessary to determine whether DGAT1 inhibition improves disease outcomes during systemic Kp infection.

Collectively, our findings identify DGAT1 as an immunometabolic regulator that links LD biogenesis, lysosomal dysfunction, mitochondrial ROS accumulation, and pyroptotic cell death in macrophages infected with a clinical cKp isolate. More broadly, this study provides mechanistic insight into how host lipid metabolism shapes macrophage fate during cKp infection and suggests that targeting DGAT1 warrants further investigation as a potential host-directed strategy for controlling excessive inflammatory responses during severe Kp infection.

## Materials and methods

### Cell culture

Murine macrophage cell line RAW264.7 (TIB-71, American Type Culture Collection) was maintained in Dulbecco’s Modified Eagle Medium (DMEM; LM001-05, Welgene, Gyeongsan, South Korea) supplemented with 10% fetal bovine serum (S001-07, Welgene) and 1% penicillin/streptomycin (15140-122, Gibco BRL, Grand Island, NY, USA). Primary bone marrow-derived macrophages (BMDMs) were isolated from 6-week-old female C57BL/6 mice and cultured in DMEM for 3–5 days in the presence of macrophage colony-stimulating factor (M-CSF; 416-ML, R&D Systems, Minneapolis, MN, USA). All animal procedures were performed in accordance with the guidelines of the Institutional Animal Care and Use Committee (IACUC) of Keimyung University School of Medicine (approval number: KM-2023-22R1) and the Korean Food and Drug Administration. All cells were incubated at 37 °C in a humidified atmosphere containing 5% CO₂.

### Bacterial strains and culture

Sixteen Kp isolates were obtained from clinical specimens at Keimyung University Dongsan Hospital. A cKp strain was isolated from a sputum sample of a hospital-acquired pneumonia patient. Strains were identified using the VITEK 2 system (bioMérieux, Lyon, France). ESBL production was confirmed via agar dilution with cefotaxime and ceftazidime plus clavulanate following CLSI guidelines. Capsular serotypes (K1, K2, K5, K20, K54, K57) were determined by PCR [42, 43]. cKp strains were grown in Luria-Bertani (LB) broth (244620, Difco, Becton Dickinson, Sparks, MD, USA) at 37 °C or on LB agar. For infection stocks, overnight cultures were diluted into fresh LB broth and grown to an OD_600_ = 0.1. Bacteria were aliquoted in LB with 15% glycerol and stored at –80 °C. CFU counts were performed before use.

### Infection

RAW264.7 cells were replated into 6-well or 24-well plates in complete DMEM medium at a density of 7 × 10^5^ cells/well and infected with the bacteria at a multiplicity of infection (MOI) of 1 or 10 for 90 min. BMDMs were replated into 24- or 96-well plates in complete DMEM medium at a density of 2 × 10^5^ cells/well and infected with the bacteria at a MOI of 10 for 90 min. Non-phagocytosed bacteria were removed by washing twice with PBS containing 100 μg/mL gentamicin (15710-072, Gibco) and 15 μg/mL polymyxin B (81271, Sigma-Aldrich, St. Louis, MO, USA). Subsequently, fresh medium without gentamicin was added for the remainder of the experiment. To impair LD biogenesis, cells were treated with 10 μM A922500 (A1737, Sigma-Aldrich) or 5 μM T863 (SML0539, Sigma-Aldrich) for 1 h prior to infection, and treatment was maintained throughout the infection period. To inhibit the mTOR signaling pathway, cells were treated with 200 nM rapamycin (553210, Sigma-Aldrich) for 30 min prior to infection. To scavenge intracellular ROS, N-acetylcysteine (NAC; 5 mM, A7250, Sigma-Aldrich) was added to the culture medium 1 h prior to infection. Cells were treated with 50 μM atglistatin (SML1075, Sigma-Aldrich) for 1 h prior to infection. Cytochalasin D (5 μM; C8273, Sigma-Aldrich) was used as a negative control for lysosomal activity assays. All inhibitors were dissolved in dimethyl sulfoxide (DMSO; D2650, Sigma-Aldrich).

### Cell counting assay

Cells were seeded in 12-well plates in complete DMEM at a density of 4 × 10^5^ cells/well and infected with the cKp at MOI 1 or 10 for 90 min. After incubation, cells were detached from each well using Accutase (SCR005, Millipore, Burlington, MA, USA). Trypan blue solution (T8154, Sigma-Aldrich) was then added to the cell suspension. The viable cell numbers were counted using a hemocytometer.

### Lactate dehydrogenase (LDH) production assay

LDH release into the culture supernatant was measured using the EZ-LDH assay kit (DG-LD01-500, DoGenBio, Seoul, South Korea), following the manufacturer’s instructions. Briefly, BMDMs or RAW264.7 cells were cultured and infected with cKp. Then, the supernatant medium (10 μL) for each sample was transferred into a 96-well plate and incubated with the LDH reaction mixture (100 μL) for 30 min in a dark room. LDH levels in the culture medium were determined by measuring the absorbance (450 nm) using the Synergy/HTX (BioTek instrument, Inc., Winooski, VT, USA).

### Flow cytometric analysis

Cell cycle analysis was performed using flow cytometry. For DNA content analysis, approximately 1 × 10^6^ cells were fixed in 80% ethanol for at least 1 h at 4°C. Ethanol-fixed cells were stained with propidium iodide (PI; P4864, Sigma-Aldrich) staining solution (50 μg/mL PI, 0.1 mg/mL RNase A, 0.1% NP-40, 0.1% trisodium citrate) for 30 min, and analyzed using a FACS analyzer (BD Biosciences, San Diego, CA, USA).

To assess intracellular LD accumulation, RAW264.7 cells and BMDMs were treated with the indicated inhibitors for 1 h prior to infection with cKp (MOI 10) for 90 min. After two washes with PBS containing gentamicin (100 μg/mL), cells were incubated in DMEM for 4 h, then stained with BODIPY 493/503 (D3922; Molecular Probes, Eugene, OR, USA) in plain DMEM at 37 °C for 30 min. Live cells were acquired using a BD FACSCanto II flow cytometer (BD Biosciences) and analyzed using BD FACSDiva software.

### Transmission electron microscopy

For transmission electron microscopy analysis, samples were sequentially fixed with 2.5% glutaraldehyde and 1% osmium tetroxide on ice for 2 h, followed by washing with PBS. The cells were then dehydrated through a graded ethanol and propylene oxide series, embedded in an Epon 812 mixture, and polymerized in an oven at 60 °C for 24 h. Ultrathin sections (70 nm thick) were obtained from the polymerized blocks, mounted on 75-mesh copper grids, counterstained with 4% uranyl acetate for 10 min and lead citrate for 7 min, and examined using a KBSI Bio-HVEM system (JEM-1400Plus at 120 kV and JEM-1000BEF at 1000 kV; JEOL, Japan).

### Immunoblotting

Cells were lysed in RIPA lysis buffer (89900, Thermo Scientific™, Waltham, MA, USA) and supernatant fractions were collected. Protein concentrations were measured using a bicinchoninic acid (BCA) protein assay kit (23225, Pierce, Rockford, IL, USA), and equal amounts of proteins (50 μg/lane) were separated by 13% sodium dodecyl-sulfate polyacrylamide gel electrophoresis and transferred to nitrocellulose membranes (10600002, GE Healthcare Life Sciences, Pittsburgh, PA, USA), followed by incubation with specific antibodies. The anti-caspase-1 antibody (3019-100; BioVision, Milpitas, CA, USA), anti-DGAT1 antibody (NB110-41487, Novus Biologicals, Centennial, CO, USA), anti-phospho-mTOR (Ser2448) (#2971, Cell Signaling Technology, Danvers, MA, USA), and anti-phospho-p70S6K (Thr421/Ser424) (#9204, Cell Signaling Technology) were used as primary antibodies. These antibodies were used at a dilution of 1:1000. The anti-ACTB antibody (A5441) was acquired from Sigma-Aldrich and used at a dilution of 1:5000. Secondary antibodies (anti-rabbit IgG [1:5000 diluted; 111-035-045] and anti-mouse IgG [1:5000 diluted; 115-035-062]) were purchased from Jackson ImmunoResearch Laboratories, Inc. (West Grove, PA, USA). Bands were detected using the Immobilon Western Chemiluminescent HRP Substrate (WBKLS0500, EMD Millipore, Darmstadt, Germany).

### RNA isolation and quantitative real-time polymerase chain reaction (qRT-PCR)

Total RNA was extracted from infected cells using TRIzol (15596-026; Thermo Fisher Scientific, Waltham, MA, USA), following the manufacturer’s instructions. For miRNA analysis, total miRNA was isolated using the miRNeasy Mini Kit (217004, Qiagen, Hilden, Germany) according to the manufacturer’s instructions. Then, RNA was reverse-transcribed to complementary DNA using the reverse-transcriptase premix (EBT-1515; Elpis Biotech, Daejeon, South Korea). qPCR was performed on a qTOWER3 thermal cycler (Analytik Jena, Jena, Germany) with THUNDERBIRD SYBR qPCR Mix (QPS-201, TOYOBO, Osaka, Japan). Relative gene expression was calculated by the 2^–ΔΔCt^ method and normalized to *β-actin*. qRT-PCR analysis of miR-155-5p was performed using the mmu-miR-155-5p primer set (YP02119303, Qiagen), and expression levels were normalized to U6 (YD00699002, Qiagen). The primer sequences (mouse) were as follows: *Acaca* forward: 5’-GATATCCCAGAGATGTTTCGGC-3’, reverse: 5’-GTCAGCATGTCAGAAGGCAGAG-3’; *Scd1* forward: 5’-CCGAAGTCCACGCTCGAT-3’, reverse: 5’-TGGAGATCTCTTGGAGCATGTG-3’; *Fasn* forward: 5’-CACAGTGCTCAAAGGACATGCC-3’, reverse: 5’-CACCAGGTGTAGTGCCTTCCTC-3’; *Il1b* forward: 5’-TACGGACCCCAAAAGATGA-3’, reverse: 5’-TGCTGCTGCGAGATTTGAAG-3’; *Nlrp3* forward: 5’-TCACAACTCGCCCAAGGAGGAA-3’, reverse: 5’-AAGAGACCACGGCAGAAGCTAG-3’; and *β-actin* forward: 5’-CCACCATGTACCCAGGCATT-3’, reverse: 5’-AGGGTGTAAAACGCAGCTCA-3’.

### Small interfering RNA transfection

For transient silencing of *Dgat1* in RAW264.7 murine macrophage cells, *Dgat1* siRNA (#sc-40488) and a scrambled siRNA (#sc-37007) were obtained from Santa Cruz Biotechnology, Inc (Dallas, TX, USA). All siRNAs were transfected using Lipofectamine^®^ 2000 Reagent (11668027, Invitrogen, Carlsbad, CA, USA). RAW264.7 cells were seeded in 24-well plates at a density of 20 × 10^4^ cells per well and transfected with the 100 nM siRNAs using Lipofectamine^®^ 2000 Reagent (Invitrogen). After 4 h of transfection, the medium was replaced with complete DMEM, and cells were incubated for an additional 24 h. The transfected cells were then infected with cKp for 8 h and harvested for cell death analysis by PI staining and flow cytometry.

### miRNA mimic and inhibitor transfection

For transient modulation of miR-155-5p expression in RAW264.7 murine macrophage cells and BMDMs, miR-155-5p mimic (MIMAT0000165), miR-155-5p inhibitor (MIMAT0000165), miRNA mimic negative control, and miRNA inhibitor negative control were purchased from Genolution (Seoul, South Korea). Cells were seeded in 24-well plates at a density of 20 × 10^4^ cells per well and transfected with 100 nM miRNA mimic or inhibitor using Lipofectamine® 2000 Reagent (Invitrogen). After 4 h of transfection, the medium was replaced with complete DMEM, and cells were incubated for an additional 24 h prior to cKp infection.

### Enzyme-linked immunosorbent assay (ELISA)

BMDMs were cultured in 24-well culture plates at a density of 2 × 10^5^ cells per well. Supernatants were collected after overnight culture and centrifuged at 2000 × *g* for 5 min at 4 °C to remove cell debris. Culture supernatants were assayed for IL-1β and IL-18 using the mouse IL-1β/IL-1F2 Quantikine ELISA Kit (MLB00C, R&D Systems, Minneapolis, MN, USA) and the mouse IL-18 ELISA Kit (ab216165, Abcam, Cambridge, UK) according to the manufacturer’s instructions.

### Caspase-1 activity assay

Caspase-1 activity was detected using the FAM-FLICA Caspase-1 (YVAD) Assay Kit (#98; ImmunoChemistry Technologies, Davis, CA, USA) according to the manufacturer’s instructions. Murine macrophages on coverslips were infected and stained with a staining solution at 37 °C for 1 h. After washing with 1× apoptosis wash buffer, cells were counterstained with 4′,6-diamidino-2-phenylindole (DAPI, D9542; Sigma-Aldrich) at 37 °C for 10 min, fixed, and imaged using a confocal laser microscope (TCS SP8; Leica Microsystems, Wetzlar, Germany) and Image J software (National Institutes of Health, Bethesda, MD, USA).

### Detection of cathepsin B activation

The infected cells were stained using the Magic Red Cathepsin B assay kit (#938; ImmunoChemistry Technologies) at 37 °C for 1 h, and nuclei were stained with DAPI at 37 °C for 10 min. Cells were fixed with 4% paraformaldehyde (DGB5004, DoGenBio, Seoul, South Korea), and images were visualized by a confocal laser microscope (TCS SP8).

### Immunofluorescence and analysis

For LD quantification, infected cells were fixed with 4% paraformaldehyde (DoGenBio) for 15 min and then permeabilized with 0.25% Triton X-100 (T8787, Sigma-Aldrich) for 10 min. Next, cells were incubated with BODIPY 493/503 (D3922; Molecular Probes), according to the manufacturer’s instructions. Nuclei were stained with DAPI for 5 min at room temperature (RT). Immunofluorescence images were observed and analyzed using the confocal laser microscope (TCS SP8) and ImageJ software.

For NLRP3 staining, cells were fixed with 4% paraformaldehyde (DoGenBio) for 15 min and then permeabilized with 0.25% Triton X-100 (Sigma-Aldrich) for 10 min. Next, cells were incubated with anti-NLRP3 (1:400 diluted; 15101; Cell Signaling) primary antibodies overnight at 4 °C. Then, cells were washed with PBS followed by an incubation with secondary Alexa Fluor 488 goat anti-rabbit IgG (H + L) (1:400 diluted; A-11034, Invitrogen) at RT for 2 h. Nuclei were stained with DAPI for 5 min at RT. Lysosomes were stained with LysoTracker Yellow-HCK-123 (L12491; Thermo Fisher Scientific), according to the manufacturer’s instructions. Immunofluorescence images were observed and analyzed using the confocal laser microscope (TCS SP8) and ImageJ software.

### TG level measurement

According to the manufacturer’s instructions, levels of TG in macrophage lysate were measured using EZ-Triglyceride Quantification Assay kits (DG-TGC100, DoGenBio, Seoul, South Korea). Briefly, RAW264.7 cells were cultured and infected with cKp. Then, the cell lysates (50 μL) for each sample were transferred into a 96-well plate and incubated with the TG reaction mixture (50 μL) at RT for 30 min in a dark room. Cellular TG levels were determined by measuring fluorescence intensity (Ex/Em = 535/595 nm) using a Synergy/HTX (BioTek Instrument, Inc).

### ROS production

Intracellular ROS levels were measured using 2’,7’-dichlorodihydrofluorescein diacetate (H₂DCFDA; D6883, Sigma-Aldrich). Cells were stained with 10 μM H₂DCFDA for 30 min at 37 °C, trypsinized, resuspended in PBS, and analyzed using a BD FACSCanto™ II flow cytometer (BD Biosciences) at excitation/emission wavelengths of 488/520 nm. Data were analyzed using BD FACSCanto™ software (version 6.0). Intracellular ROS were also quantified using a microplate reader (Infinite M200 Pro; Tecan Group Ltd., Männedorf, Switzerland) at excitation/emission wavelengths of 488/520 nm, with cells seeded in 96-well black plates and stained with 10 μM H₂DCFDA for 30 min at 37 °C. Mitochondrial ROS were assessed using MitoSOX™ Red (M36008; Invitrogen), and lipid peroxidation was evaluated using BODIPY™ 581/591 C11 (D3861; Thermo Fisher Scientific). For both stainings, cells were incubated with the respective dye for 30 min at 37 °C, trypsinized, and resuspended in PBS. Fluorescence was measured using a BD FACSCanto™ II flow cytometer (BD Biosciences). MitoSOX fluorescence was detected in the PE channel, and oxidized C11-BODIPY 581/591 fluorescence was detected in the FITC channel.

### Library preparation and sequencing

For control and test RNAs, the construction of the library was performed using the NEBNext Multiplex Small RNA Library Prep kit (E7300, New England BioLabs, Inc., USA) according to the manufacturer’s instructions. Briefly, for library construction, total RNA from each sample was used to ligate the adaptors, and then cDNA was synthesized using reverse-transcriptase with adaptor-specific primers. PCR was performed for library amplification, and libraries were cleaned up using QIAquick PCR Purification Kit (28104, Qiagen) and polyacrylamide gel electrophoresis (PAGE) gel. The yield and size distribution of the small RNA libraries were assessed using the Agilent 2100 Bioanalyzer instrument with the High-sensitivity DNA Assay kit (5067-4626, Agilent Technologies, Inc., Santa Clara, CA, USA). High-throughput sequences were performed on the NextSeq550 system (Illumina, San Diego, CA, USA) using single-end 75 sequencing.

### sgRNA design for *Dgat1* targeting

Single guide RNA (sgRNA) sequences targeting the mouse *Dgat1* gene were designed using the Benchling CRISPR design platform. sgRNAs were selected to target early coding exons of *Dgat1* to maximize the probability of generating frameshift mutations through non-homologous end joining (NHEJ). Candidate sgRNAs were prioritized based on predicted on-target efficiency and minimal off-target scores provided by Benchling. The sgRNA protospacer sequences were as follows: sgRNA#1_top: 5’-CACCGATACCCGGGACAAAGACGGG-3’, sgRNA#1_bottom: 5’-AAACCCCGTCTTTGTCCCGGGTATC-3’.

### Construction of CRISPR/Cas9 plasmids and transfection of RAW264.7 cells

sgRNA oligonucleotides were synthesized, annealed, and cloned into the PX330-derived SpCas9-2A-mCherry vector (#98750, Addgene plasmid, Watertown, MA, USA) using *Bbs*I-based Golden Gate cloning. Ligation reactions were transformed into *Escherichia coli*, and positive clones were screened by colony PCR and confirmed by Sanger sequencing to verify correct sgRNA insertion and orientation. RAW264.7 cells were seeded one day prior to transfection to achieve approximately 70% confluency at the time of transfection. Cells were transfected with the sgRNA–Cas9 expression plasmid using Lipofectamine 3000 (L3000015, Thermo Fisher Scientific) according to the manufacturer’s instructions. Transfection complexes were prepared in Opti-MEM Reduced Serum Medium (31985-070, Gibco) and applied to cells under serum-containing conditions. Cells were incubated for 48 h to allow Cas9 expression and genome editing.

### FACS and enrichment of edited cells

At 24 h post-transfection, RAW264.7 cells were harvested and subjected to FACS using a CytoFLEX SRT cell sorter (Beckman Coulter, Brea, CA, USA). Cells expressing mCherry fluorescence, indicative of Cas9 expression, were gated and sorted under sterile conditions. Sorted mCherry-positive cells were pooled, replated onto 100 mm culture dishes containing complete growth medium, and allowed to recover and expand under standard culture conditions prior to clonal isolation. Individual colonies were manually picked under microscopic guidance and transferred to separate wells of 96-well plates for clonal expansion. Each clone was sequentially expanded for downstream genotyping and validation analyses.

### Genotyping and identification of *Dgat1* heterozygous knockout clones

Genomic DNA was isolated from expanded clonal cell populations using the GenEx™ Cell SV Kit (106-101, GeneAll Biotechnology, Seoul, South Korea) according to the manufacturer’s instructions. The genomic region flanking the CRISPR/Cas9 target site in the *Dgat1* gene was amplified by PCR using locus-specific primers. PCR amplicons were subjected to Sanger sequencing, and sequencing chromatograms were analyzed to detect insertion/deletion (indel) mutations at the expected Cas9 cleavage site. In representative clones, one allele harbored a 72 bp deletion, which introduced a premature stop codon at amino acid position 420, while the second allele retained the wild-type sequence. Clones exhibiting overlapping sequencing peaks consistent with the presence of one wild-type allele and one indel-containing allele were classified as *Dgat1* heterozygous knockout (*Dgat1*^+^/^−^) clones.

### Data analysis

Sequence reads were mapped by the bowtie2 software tool in order to obtain a BAM file. A mature miRNA sequence is used as a reference for mapping. Read counts mapped on mature miRNA sequences were extracted from the alignment file using bedtools v2.25.0 [44] and Bioconductor [45], which uses R statistical programming language [46]. Read counts were used in order to determine the expression level of miRNAs. The CPM + TMM normalization method was used for comparison between samples. For the miRNA target study, miRWalk 2.0 [47] was performed. Functional gene classification was performed by DIANA [48].

### Statistical analysis

Data are presented as mean ± standard deviation and were obtained from three independent experiments. Data normality was assessed using the Shapiro-Wilk test. Homogeneity of variance between groups was assessed using Levene’s test prior to statistical comparisons. Differences between two groups were analyzed using Student’s *t* test, and differences among more than two groups were analyzed using one-way ANOVA followed by Tukey’s post-hoc test. Statistical analyses were performed, and graphs were generated using GraphPad Prism 7.0 software for Windows (GraphPad Software Inc., La Jolla, CA, USA). *P*-values < 0.05, < 0.01, and < 0.001 were considered statistically significant.

## Supplementary information

Supplementary Information

Supplementary figure_Uncropped Western Blots

## Data Availability

All data generated during this study are included in this article and supplementary data.
