# Supplementary material for: DGAT1 regulates macrophage pyroptosis during Klebsiella pneumoniae infection
Source: Cell Death Discov. 2026 Aug 11;12:446. doi: 10.1038/s41420-026-03272-y (PMC13740289; doi:10.1038/s41420-026-03272-y)
Supplement: Supplementary file 2 [file 41420_2026_3272_MOESM2_ESM.pdf]

Supplementary Information

DGAT1 Regulates Macrophage Pyroptosis during *Klebsiella pneumoniae* Infection

Hui-Jung Jung, Seong Hun Jeong, Sang-Hee Lee, Hyowon Hong, Yong-Uk Lee,

Miri Hyun, Ji Yeon Lee, Won-Ki Baek, Jin Kyung Kim, Hyun ah Kim

This file contains Supplementary Figures S1–S6

Supplementary Figure 1

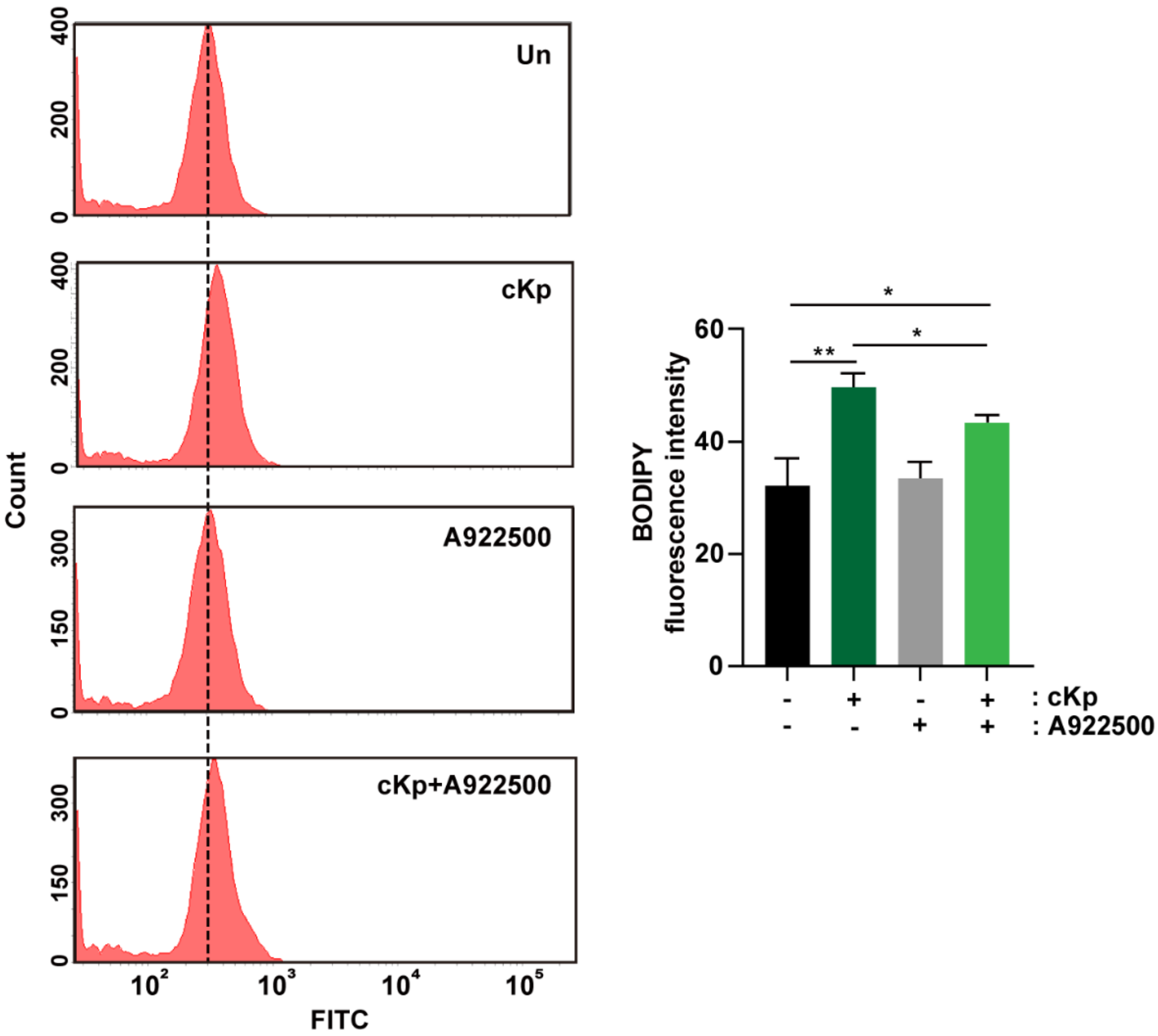

Supplementary Figure. 1 DGAT1 inhibition attenuates cKp-induced LD accumulation in BMDMs.

BMDMs were pretreated with or without A922500 (10 μM) for 1 h and then infected with cKp (MOI 1) and incubated for 24 h. LD formation was assessed by BODIPY 493/503 fluorescence intensity using flow cytometry. Representative flow cytometry histograms (left) and quantification are shown (right). All quantitative data are expressed as the mean ± SD of three independent experiments (n = 3). Un, uninfected. *P < 0.05 and **P < 0.01.

Supplementary Figure 2

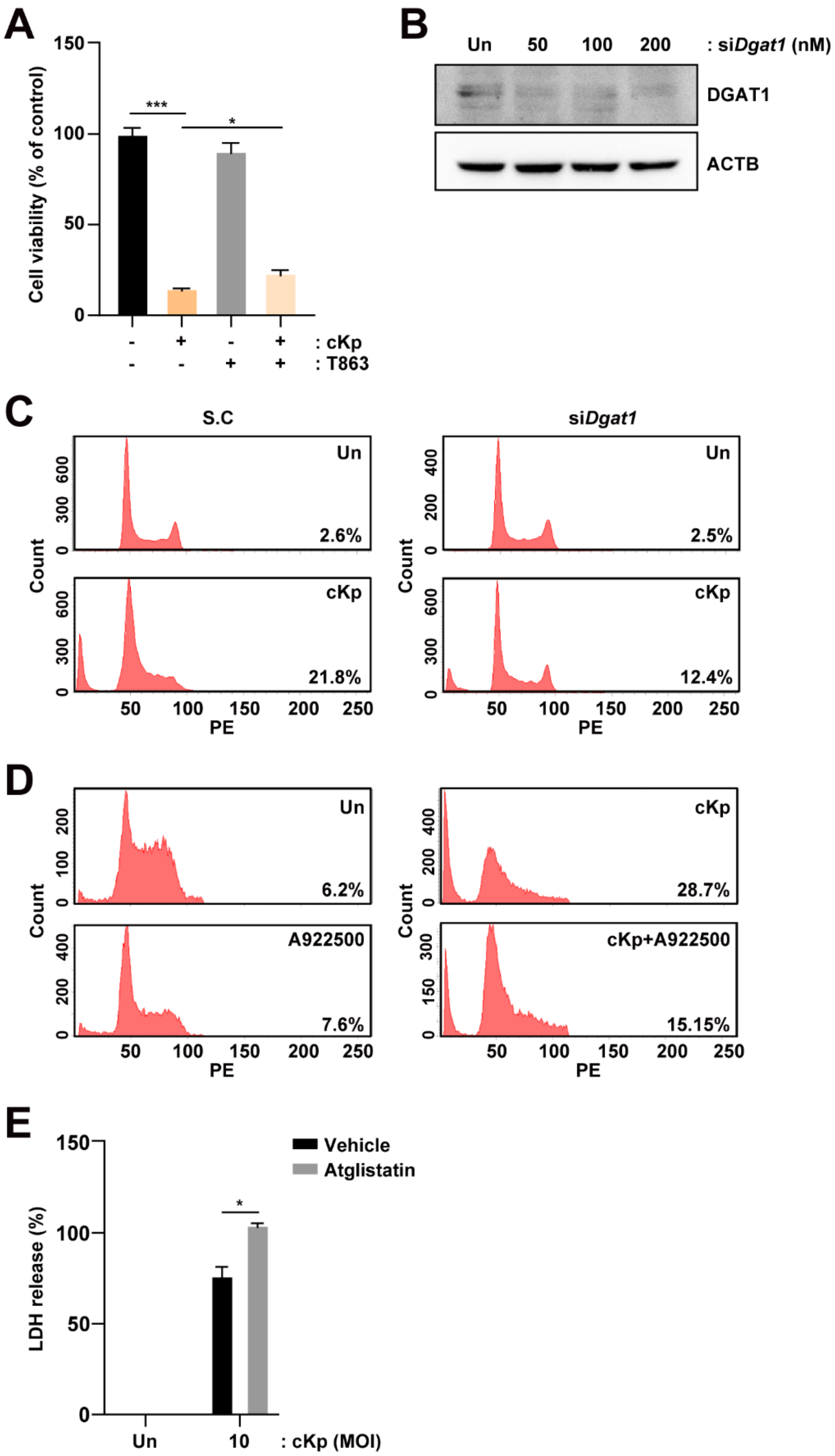

Supplementary Figure. 2 Pharmacological and genetic inhibition of DGAT1 modulates cell death in cKp-infected macrophages.

(A) RAW264.7 cells were pretreated with T863 (5 μM) for 1 h, followed by infection with cKp (MOI 10) for 16 h. Cell death was assessed by cell counting using trypan blue staining. (B) RAW264.7 cells were transfected with increasing concentrations of *Dgat1* siRNA for 24 h, and DGAT1 protein expression was analyzed by immunoblotting. ACTB was used as a loading control. (C) Representative flow cytometry histograms related to Fig. 2B. (D) Representative flow cytometry histograms related to Fig. 2C. (E) RAW264.7 cells were pretreated with the lipolysis inhibitor atglistatin (50 μM) for 1 h, followed by infection with cKp (MOI 10) for 16 h. Cell death was assessed by measuring LDH release. All quantitative data represent the mean ± SD from three independent experiments (n = 3). Un, uninfected; S.C, scrambled control. *P < 0.05 and ***P < 0.001.

Supplementary Figure 3

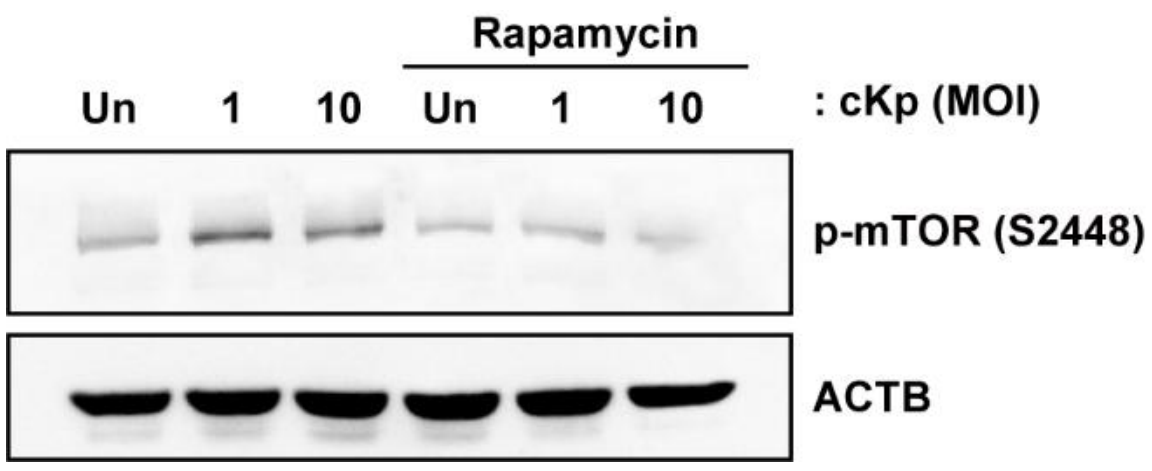

Supplementary Figure. 3 Rapamycin inhibits mTOR phosphorylation in cKp-infected macrophages.

RAW264.7 cells were pretreated with or without rapamycin (200 nM) for 30 min and then infected with cKp at the indicated MOIs and incubated for 8 h. Cell lysates were subjected to immunoblotting using antibodies against p-mTOR (S2448), with ACTB as a loading control. Un, uninfected.

Supplementary Figure 4

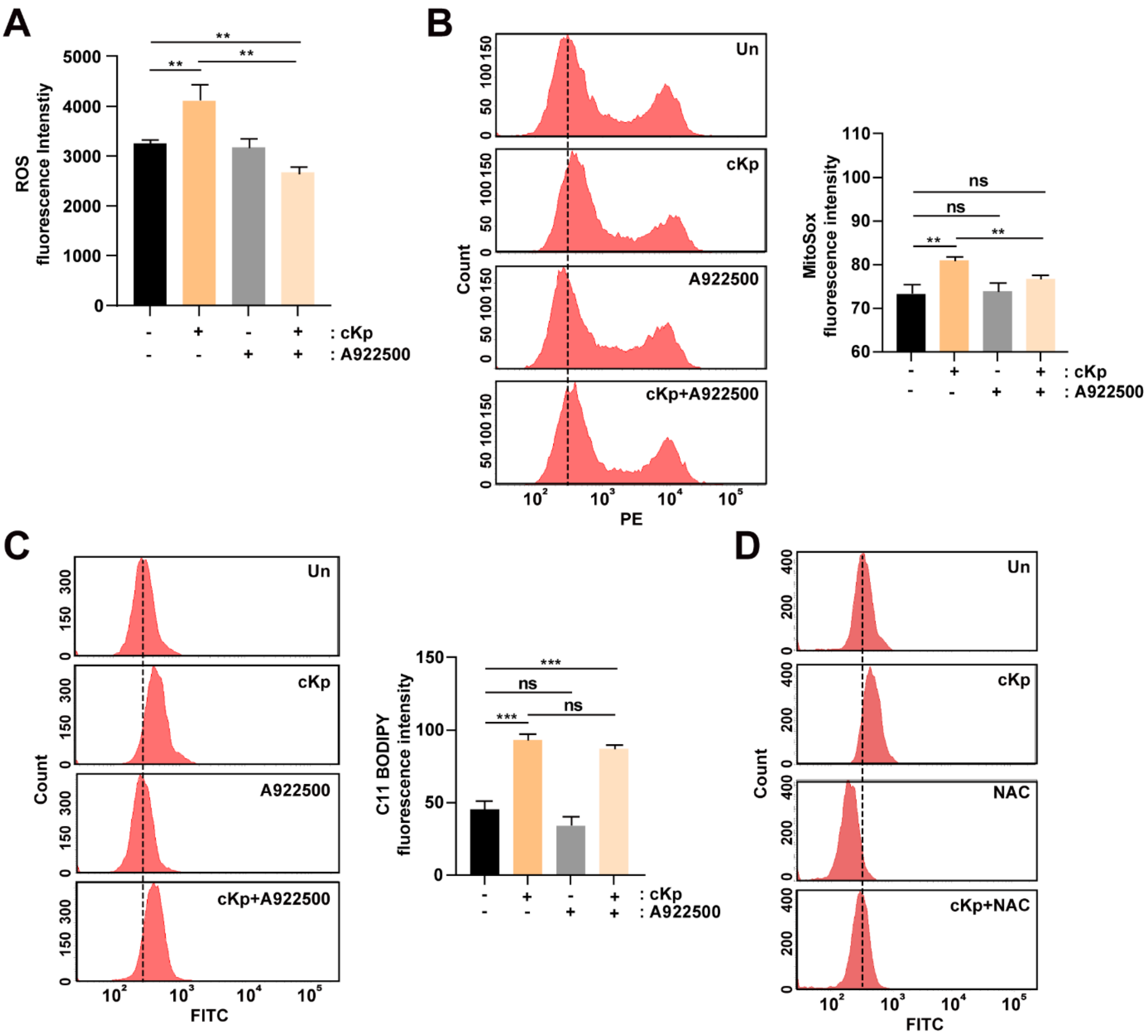

Supplementary Figure. 4 DGAT1 inhibition reduces LD accumulation and mitochondrial ROS production, but does not affect lipid peroxidation, in cKp-infected macrophages.

(A) BMDMs were pretreated with or without A922500 (10 μM) for 1 h and then infected with cKp (MOI 10) and incubated for 4 h. Intracellular ROS levels were quantified using H_2DCFDA and measured by a microplate reader at excitation/emission wavelengths of 488/520 nm. (B, C) RAW264.7 cells were pretreated with or without A922500 (10 μM) for 1 h and then infected with cKp (MOI 10) and incubated for 4 h. Mitochondrial ROS were assessed using MitoSOX™ Red staining (B) and lipid peroxidation was

evaluated using BODIPY™ 581/591 C11 staining (C) by flow cytometry. Representative flow cytometry histograms (left) and quantification are shown (right). (D) Representative flow cytometry histograms related to Fig. 5D. All quantitative data represent the mean ± SD from three independent experiments (n = 3). Un, uninfected; ns, not significant. **P < 0.01 and ***P < 0.001.

Supplementary Figure 5

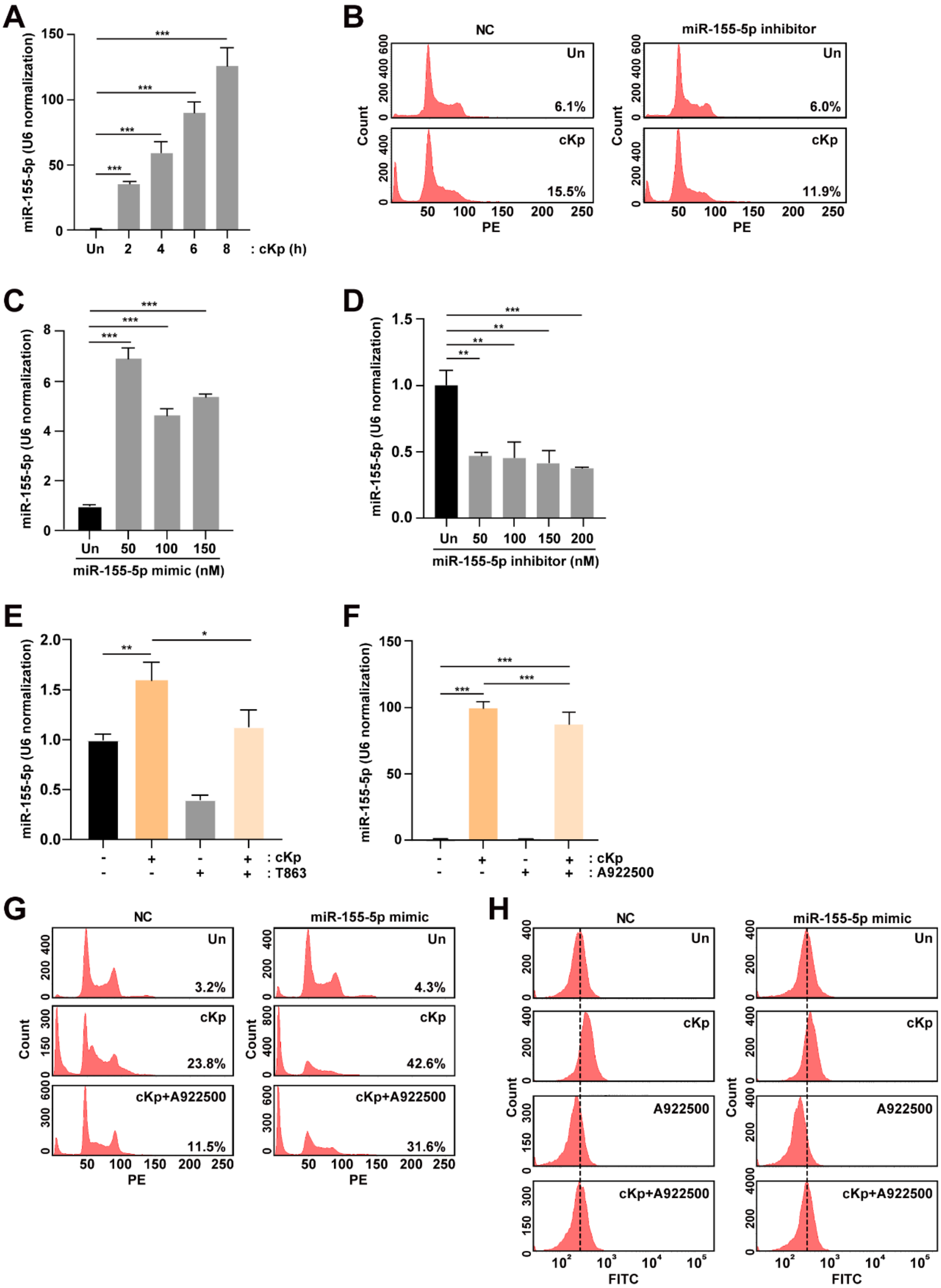

Supplementary Figure. 5 DGAT1 inhibition suppresses miR-155-5p expression and attenuates miR-155-5p-driven pyroptosis and LD accumulation during cKp infection.

(A) BMDMs were infected with cKp (MOI 10) and incubated for the indicated times. Expression levels of miR-155-5p were determined by qRT-PCR. (B) Representative flow cytometry histograms related to Fig. 6E. (C, D) RAW264.7 cells were transfected with increasing concentrations of miR-155-5p mimic (50, 100, or 150 nM) (C) or miR-155-5p inhibitor (50, 100, 150, or 200 nM) (D) for 24 h. Expression levels of miR-155-5p were determined by qRT-PCR. (E) RAW264.7 cells were pretreated with or without T863 (5 μM) for 1 h and then infected with cKp (MOI 10) and incubated for 8 h. Expression levels of miR-155-5p were determined by qRT-PCR. (F) BMDMs were pretreated with or without A922500 (10 μM) for 1 h and then infected with cKp (MOI 10) and incubated for 8 h. Expression levels of miR-155-5p were determined by qRT-PCR. (G) Representative flow cytometry histograms related to Fig. 6H. (H) Representative flow cytometry histograms related to Fig. 6J. All quantitative data are expressed as the mean ± SD of three independent experiments ($n = 3$). Un, uninfected; NC, negative control. *$P < 0.05$, **$P < 0.01$, ***$P < 0.001$.

Supplementary Figure 6

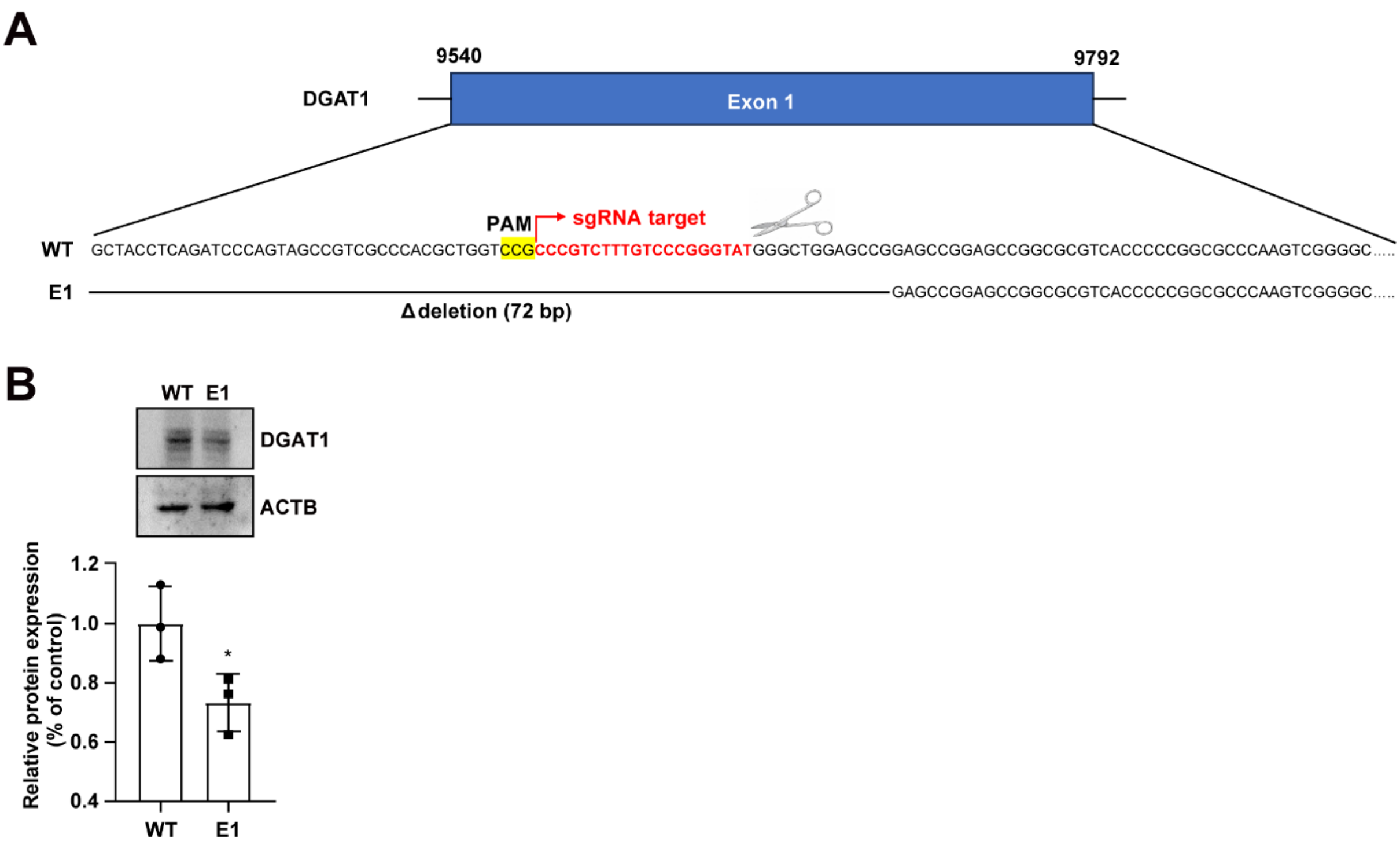

Supplementary Figure. 6 Construction and validation of the CRISPR/Cas9 system.

(A) Schematic of the *Dgat1* gene and Cas9/gRNA target site. *Dgat1* heterozygous knockout cell line (E1), targeting exon 1 of *Dgat1* using the CRISPR/Cas9 system. (B) Immunoblot analysis of DGAT1 protein levels in WT and *Dgat1* heterozygous knockout (E1) RAW264.7 cells. All quantitative data represent the mean ± SD from three independent experiments (n = 3). sgRNA, single guide RNA; PAM, protospacer adjacent motif; WT, wild type. *P < 0.05.
